# A systematic review, meta-analysis, and meta-regression of the prevalence of self-reported disordered eating and associated factors among athletes worldwide

**DOI:** 10.1186/s40337-024-00982-5

**Published:** 2024-02-07

**Authors:** Hadeel A. Ghazzawi, Lana S. Nimer, Areen Jamal Haddad, Omar A. Alhaj, Adam T. Amawi, Seithikurippu R. Pandi-Perumal, Khaled Trabelsi, Mary V. Seeman, Haitham Jahrami

**Affiliations:** 1 Department of Nutrition and Food Technology, School of Agriculture, The University of Jordan, Amman, Jordan; 2 Department of Nutrition, Faculty of Pharmacy and Medical Sciences, University of Petra, Amman, Jordan; 3 Department of Exercise Science and Kinesiology, School of Sport Science, The University of Jordan, Amman, Jordan; 4 Saveetha Medical College and Hospitals, Saveetha Institute of Medical and Technical Sciences, Saveetha University, Chennai, Tamil Nadu India; 5 High Institute of Sport and Physical Education of Sfax, University of Sfax, 3000 Sfax, Tunisia; 6 Research Laboratory: Education, Motricity, Sport and Health, University of Sfax, EM2S, LR19JS013000 Sfax, Tunisia; 7 Department of Psychiatry, University of Toronto, Toronto, ON M5S 1A8 Canada; 8 Ministry of Health, Manama, Bahrain; 9 Department of Psychiatry, College of Medicine and Medical Sciences, Arabian Gulf University, Manama, Bahrain; 10 Division of Research and Development, Lovely Professional University, Phagwara, Punjab 144411 India

**Keywords:** Anorexia, Aerobic energy, Athletes, Eating disorders, Sport type, World region

## Abstract

**Background:**

The purpose of this meta-analysis was to provide a pooled prevalence estimate of self-reported disordered eating (SRDE) in athletes based on the available literature, and to identify risk factors for their occurrence.

**Methods:**

Across ten academic databases, an electronic search was conducted from inception to 7th January 2024. The proportion of athletes scoring at or above predetermined cutoffs on validated self-reporting screening measures was used to identify disordered eating (DE). Subgroup analysis per country, per culture, and per research measure were also conducted. Age, body mass index (BMI), and sex were considered as associated/correlated factors.

**Results:**

The mean prevalence of SRDE among 70,957 athletes in 177 studies (132 publications) was 19.23% (17.04%; 21.62%), *I*^*2*^ = 97.4%, τ^2^ = 0.8990, Cochran's Q p value = 0. Australia had the highest percentage of SRDE athletes with a mean of 57.1% (36.0%-75.8%), while Iceland had the lowest, with a mean of 4.9% (1.2%-17.7%). The SRDE prevalence in Eastern countries was higher than in Western countries with 29.1% versus 18.5%. Anaerobic sports had almost double the prevalence of SRDE 37.9% (27.0%-50.2%) compared to aerobic sports 19.6% (15.2%-25%). Gymnastics sports had the highest SRDE prevalence rate, with 41.5% (30.4%-53.6%) while outdoor sports showed the lowest at 15.4% (11.6%-20.2%). Among various tools used to assess SRDE, the three-factor eating questionnaire yielded the highest SRDE rate 73.0% (60.1%-82.8%). Meta-regression analyses showed that female sex, older age, and higher BMI (all p < 0.01) are associated with higher prevalence rates of SRDE.

**Conclusion:**

The outcome of this review suggests that factors specific to the sport affect eating behaviors throughout an athlete's life. As a result, one in five athletes run the risk of developing an eating disorder. Culture-specific and sport-specific diagnostic tools need to be developed and increased attention paid to nutritional deficiencies in athletes.

**Supplementary Information:**

The online version contains supplementary material available at 10.1186/s40337-024-00982-5.

## Abstract (plain language summary)

Disordered eating (DE) refers to eating behaviors that limit food choices, reduce or exaggerate food intake, cause physical discomfort, create a sense of loss of control, or lead to negative emotions like shame or guilt. The DE label does not signify the presence of a diagnosed mental health disorder, but, rather, describes self-reported, regular eating patterns that psychiatrists consider to fall into the at-risk category for an eating disorder. In this review, we performed a search of academic databases to find all relevant studies that measured the frequency of self-reported DE in athletes. We analyzed 177 studies involving over 70,000 athletes in total. In this study, DE was defined as a score above cut-off on validated screening tests for problematic eating behaviors. Our goal was to estimate the prevalence of DE in athletes globally and to determine the factors that increase risk. We found that approximately 1 in 5 athletes (19%) endorsed DE behaviors such as, among others, restrictive dieting, bingeing, and purging. These behaviors were seen most commonly in indoor sports like gymnastics and less commonly in the context of outdoor sports. Rates were highest in female athletes, older athletes, athletes with high body mass index (BMI) scores, and those from Eastern countries and cultures. Australia had the highest rates (over 50%), while rates were lowest in Iceland. We suggest the development of screening tests specifically tailored and applicable to athletes. In addition, we recommend raising public awareness of the health effects of nutrition in sports.

**Supplementary Information:**

The online version contains supplementary material available at 10.1186/s40337-024-00982-5.

## Introduction

Eating disorders (EDs) are serious, all too often chronic, mental illnesses that usually begin in adolescence or young adulthood [1, 2]. Approximately thirty million people globally suffer from EDs, disorders that are frequently misdiagnosed and under (ineffectively) treated [3]. There is general agreement that a variable and complicated combination of biological, psychological, social, and cultural factors cumulatively increases the risk of ED [4].

In the general adult population, anorexia nervosa (AN) and bulimia nervosa (BN) are clinically recognized EDs that, at least in part, reflect the extremes of a continuum of traits that include negative body image, food restraint, and preoccupation with weight and musculature in the formation of self-identity [5, 6]. According to longitudinal risk factor studies [5, 6], negative body image and "disordered eating" (DE), and "screening for at-risk" individuals is where this continuity assumption is most visibly present [7, 8]. Continuity assumption in this context refers to the assumption that DE lies on a continuum from normal eating to clinical disorders [9].

Negative body image and DE are the strongest predictors of the development of full-blown EDs [10]. Together, they constitute an "at risk" state [11]. While usually associated with women, and attributed to occupations such as modeling or ballet where weight must be restricted, EDs are also prevalent in males, and especially in both male and female sports [12, 13]. They are most likely to develop in athletes who compete in sports that a) promote leanness as a way to improve performance, b) divide team eligibility according to weight, or c) receive points based on appearance [14]. Athletes' risk of acquiring EDs varies according to their gender, sport, and also level of competition [15]. Self-reported measures for DE are useful as screening tools because they are inexpensive, and easy to apply [8, 16]. They help identify preclinical "at risk" DE behavior [8, 16].

In a recent published meta-analysis [5] of SRDE in university students, our research team applied the Levine and Smolak definition of DE [17, 18]. This definition consists of: a) unhealthy, maladaptive, and stress-inducing levels of negative body image, weight and shape concerns, dietary restrictions and/or binge eating [2, 17]; b) at least two of the following: individual ED symptoms such as self-induced vomiting after eating, abuse of laxatives, diuretics, diet pills, and exercise, unrealistic beauty standards, including an idealization of thinness, irrational and maladaptive beliefs about body fat and fat people, often coupled with a strong drive for thinness, relatively high levels of negative affect that is difficult to endure and manage, and harsh self-surveillance and self-criticism, often accompanied by low and unstable self-esteem [5]. Noting the lack of a previously agreed-upon definition of DE and thus the lack of research data about its point prevalence, Levine and Smolak [2]estimated their definition of DE to be present in 15–20% of the general population. They noted, however, that available studies used different definitions and different cut-off points for DE on questionnaires such as the Eating Attitudes Test (EAT-26) [2].

The connection between DE and sports, has been gaining attention over the last two decades[8]. Up to 5% of people in the community have ED, and, athletes report higher frequencies of EDs than non-athletes. In one study, when compared to non-athletes, female athletes showed a 45% higher prevalence of DE practices (*i.e.,* under the threshold for a diagnosis of full-blown ED). In male athletes, the rate was 19% higher than for non-athletes [19]. A recent article from Jordan by Ghazzawi et al., (2022) indicated that overall, 35% (1%-62%) of Jordanian athletes were at substantial risk for ED [20]. As mentioned earlier, several factors explain discrepancies among reports and account for the broad prevalence range cited by Ghazzawi et al. [20–23].

Recent studies point to the promotion of a healthy body image as a helpful foundation for addressing athletes' DE [24, 25]. Athletes may be susceptible to EDs because the prevalent idealized body image in sports emphasizes leanness or low body weight, particularly so in endurance, aesthetic, and weight class competitions [15, 24, 26]. Other contributing factors are: early sport-specific training, participation in weight-class sports, the engrained habit of frequent dieting and subsequent weight fluctuation[27], the wearing of skimpy sportswear, coaching pressure, and involvement in elite-level competition [20, 26, 28]. Female athletes are considered more vulnerable than males to concerns regarding body mass and shape; they may, therefore, use more extreme weight-control techniques than men [29].[29]Athletes who base their identity and, hence, their self-esteem, on their love of the game they play have less trouble with SRDE than those whose identity is based on their personal game skills and successes (which fluctuate). Low self-esteem is a significant risk factor leading to body image problems and subsequent ED [15].

Athletes may not, however, be more at risk for ED than the general population. Some meta-analyses [21] have shown a higher frequency of risky eating behaviors in non-athletes. Either way, the evidence for an elevated risk of EDs among athletes is not conclusive [22, 30, 31] In an attempt to help resolve the debate and to extend our previous meta-analytic reviews of SRDE in athletes [5, 6, 32], we conducted a meta-analysis of its global prevalence. To the best of our knowledge, based on searches of the literature and various registration platforms, this is the first such meta-analysis of **DE** and potential moderators in this population. In this meta-analysis, we aimed to synthesize the available literature to provide a pooled estimate of the prevalence of SRDE in athletes.

## Materials and Methods

The study's methodology was submitted to Open Science Framework (OSF; 10.17605/OSF.IO/EJ2QN) in September 2022. OSF is a freely available platform that enables researchers to share their research ideas with peers and to receive assistance over the duration of a study.

The review follows the guidelines for reporting, as recommended by the Preferred Reporting Items for Systematic Reviews and Meta-Analyses (PRISMA2020) [33]. The Meta-analysis of Observational Studies in Epidemiology (MOOSE) [34] procedure was followed in relation to the statistical analyses and to the reporting of results.

### Search strategy

In September 2022, two authors (HJ and HG) conducted an online literature review using ten databases. 1) PubMed/MEDLINE, 2) American Psychological Association PsycINFO, 3) ScienceDirect, 4) Springer, 5) EBSCOhost, 6) Embase, 7) Cumulative Index to Nursing and Allied Health Literature (CINAHL), 8) Scopus, 9) Web of Science, and 10) SportDiscus. The following keywords and lists were used in the full-text search: Athletes, gymnasts, or elite, are on List A. On List B are eating disorders, eating habits, feeding disorders, eating symptoms, eating attitudes, or eating issues. The punctuation marks (*) ensure that the phrase's reverse word order is considered in the search method. For instance, "disordered eating" and "eating disorders" are included when searching for "eating disord*."

The authors also manually searched the citation lists of included articles. The manual reference list checking helps safeguard against missing relevant literature, but incorporating as wide a range of terms as possible directly into the database searches aids the process. Meta-analyses that exclude grey literature have a higher propensity to overstate effect sizes and thus produce less accurate effect size estimations. As a result, we deliberately sought out organizational reports, unreleased studies, and research in lesser-known journals.

Three members of the team (LN, AH, and HT) independently evaluated the initial article selection. After screening titles, abstracts, and complete texts and eliminating duplicate studies, initial data were extracted, and their quality was assessed independently by two team members (LN and AH). Discussion among all team members was able to resolve any differences regarding a study's eligibility.

### Criteria for exclusion, inclusion, and eligibility (selection process)

The complete text of English-language papers on SRDE among athletes of all sports types and all ages around the globe was obtained for meta-analysis.

Inclusion criteria: 1) English language 2) original research 3) all types of sports, all ages, anywhere in the world 4) the prevalence of DE needed to be included 5) evaluation was performed by a screening tool with well-defined cut-offs for clinical diagnosis of eating disorders.

Exclusion criteria: 1) studies of current or former players who did not meet athlete criteria at the time of the study 2) mental health studies in athletes unrelated to DE 3) data unavailable even after attempt to contact authors directly 4) book chapters and review articles (systematic and meta-analysis) 5) studies in which prevalence rates were reported but only in the form of group means.

**Fig. 1:**
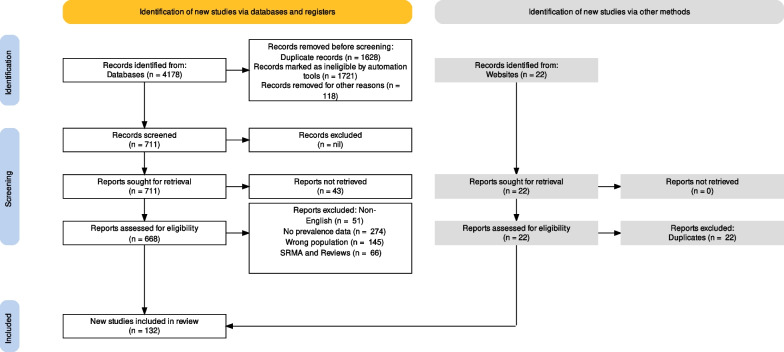
PRISMA2020 flow diagram for study selection

The PRISMA2020 process flow for research selection is shown in Fig. 1.

### Procedure (data collection process)

The 648 included papers were scanned and categorized using ASReview, an online free service that integrates digital tools (such as natural-language processing) with machine learning tools. By using a free, open-source, web-based program WebPlotDigitizer v4.5, data could be taken from plot images.

To ensure uniform data description, authors LN, AH, HT, and HG individually collected the following eight factors: author names, year of publication, country of data collection, representativeness of the sample, average BMI (kg/m^2^), gender (% of female participants), and average age (in years), and method utilized to identify SRDE presence or absence. Using the regional categories of member states created by the United Nations, this meta-analysis study included data from forty countries, which were then categorized as Eastern or Western. Eastern countries were those with emerging markets or economies in development. Western countries are defined as advanced economies with high GDP per capita, such as those in Western Europe, the U.S., Australia [35].

### Evaluation of study quality and bias risk (risk of bias assessment)

Participant selection (sample), comparability, and statistical analysis/results make up the first three items on the Newcastle–Ottawa Scale (NOS) checklist [36]. Each item in the NOS is rated on a scale of one to three (or four) stars. The highest possible rating for each study, therefore, would either be nine (for cohort studies and cross-sectional studies) or ten stars (case–control studies). Studies that score an eight are still judged to be of good quality with little chance of bias. A 5–7 score is judged to be of moderate quality with some chance of bias, and a 0–4 score is low quality with a significant chance of bias.

As noted earlier, the prevalence estimate for each study was based on a pre-defined cut-off score of a validated screening tool, that is, a continuous measure of DE risk such as the EAT-26 and Sick, Control, One stone, Fat, Food test (SCOFF) (Table 1).

**Table 1:** Characteristics of the studies involved in the systematic review and meta-analysis about the prevalence of disordered eating in Athletes

SN	REF	Study	Country	Sample Characteristics	Design	Measure
1	[59]	Abbott et al., 2021	Multiple	Female sex = 30.8%; mean age 22 years; mean BMI NR Kg/m^2^	Cross-sectional study	EAT-26
2	[46]	Akesdotter et al., 2022	Sweden	Female sex = 68%; mean age 23.5 years; mean BMI NR Kg/m^2^	Cohort study	RD
3	[48]	Al-Jumayan et al., 2021	Saudi Arabia	Female sex = 48.9%; mean age 31 years; mean BMI NR Kg/m^2^	Cross-sectional study	EAT-26
4	[60]	Alwan et al., 2022	UK	Female sex = 100%; mean age 29 years; mean BMI NR Kg/m^2^	Cross-sectional study	EAT-26
5	[61]	Anderson and Petrie 2012	USA	Female sex = 100%; mean age 22 years; mean BMI NR Kg/m^2^	Cross-sectional study	QEDD
6	[62]	Armento et al., 2023	Colorado	Female sex = 70%; mean age 40.9 years; mean BMI 22.8 kg/m^2^	Cross-sectional study	EDEQ
7	[63]	Barrack et al., 2008	USA	Female sex = 100%; mean age 22 years; mean BMI NR Kg/m^2^	Cross-sectional study	EDEQ
8	[64]	Barrack et al., b 2023	USA	Female sex = 100%; mean age 15.7 years; mean BMI 22.0 kg/m^2^	Cross-sectional study	EDEQ
9	[65]	Beals and Hill, 2006	USA	Female sex = 100%; mean age 19.5 years; mean BMI 22.9 kg/m^2^	Cross-sectional study	EDEQ
10	[66]	Beals and Manore 2002	USA	Female sex = 100%; mean age 19 years; mean BMI 22.5 kg/m^2^	Cross-sectional study	M1 = EAT-26 M2 = EDI-3
11	[67]	Beals, 2002	USA	Female sex = 100%; mean age 15.8 years; mean BMI 22.2 kg/m^2^	Cross-sectional study	EDI
12	[68]	Beekley et al., 2009	USA	Female sex = 14%; mean age 22 years; mean BMI NR Kg/m^2^	Cross-sectional study	EAT-26
13	[69]	Borgelt and Burmeister 2022	USA	Female sex = 100%; mean age 22 years; mean BMI 23.26 kg/m^2^	Cross-sectional study	EAT-26
14	[70]	Borowiec et al., b 2023	Poland	Female sex = NR%; mean age 23.2 years; mean BMI NR Kg/m^2^	Cross-sectional study	EAT-26
15	[71]	Brook et al., a 2019	Multiple	Female sex = 42.3%; mean age 31.7 years; mean BMI NR Kg/m^2^	Cohort study T1 & T2	EDEQ
16	[47]	Brown et al., 2014	USA	Female sex = 100%; mean age 22 years; mean BMI 20.88 kg/m^2^	Cross-sectional study	RD
17	[72]	Brown et al., 2020	USA	Female sex = 100%; mean age 20.15 years; mean BMI 23.29 kg/m^2^	Cohort study	EAT-26
18	[73]	Burrows et al., 2007	Canada	Female sex = 100%; mean age 31.1 years; mean BMI NR Kg/m^2^	Cross-sectional study	SIAB-S
19	[74]	Byrne and McLean 2002	Australia	Female sex = 59%; mean age 19.55 years; mean BMI 21.25 kg/m^2^	Cross-sectional study	EDI-2
20	[75]	Carvalhais et al., 2019	Portugal	Female sex = 100%; mean age 20.8 years; mean BMI NR Kg/m^2^	Cross-sectional study	EDEQ
21	[76]	Chatterton and Petrie 2013	USA	Female sex = 0%; mean age 19.9 years; mean BMI 24.3 kg/m^2^	Cross-sectional study	QEDD
22	[77]	Checa Olmos et al., b 2023	Spain	Female sex = 35.9%; mean age 14.1 years; mean BMI NR Kg/m^2^		SCOFF
23	[78]	Cobb et al., 2003	USA	Female sex = 100%; mean age 21.75 years; mean BMI 21.35 kg/m^2^	Cross-sectional study	EDI
24	[79]	Coelho et al., a 2013	Brazil	Female sex = 100%; mean age 14.77 years; mean BMI NR Kg/m^2^	Cross-sectional study	M1 = EAT-26 M2 = BITE M3 = BSQ
25	[80]	Cox et al., 1997	USA	Female sex = 92%; mean age 19.3 years; mean BMI NR Kg/m^2^	Cross-sectional study	EAT-26
26	[81]	De Borja et al., a 2021	USA	Female sex = 100%; mean age 19.8 years; mean BMI 22.86 kg/m^2^	Cross-sectional study	M1 = BEDA-Q M2 = RD M3 = ESP
27	[82]	Dervish et al., b 2023	USA	Female sex = 100%; mean age NR years; mean BMI NR Kg/m^2^	Cross-sectional study	FAST
28	[83]	Devrim et al., 2018	Turkey	Female sex = 0%; mean age 28.25 years; mean BMI NR Kg/m^2^	Cross-sectional study	EAT-40
29	[84]	Doyle-Lucas et al., 2010	USA	Female sex = 100%; mean age 24.3 years; mean BMI 18.9 kg/m^2^	Cross-sectional study	EAT-26
30	[85]	Escobar-Molina et al., 2015	Spain	Female sex = 46%; mean age 22 years; mean BMI NR Kg/m^2^	Cross-sectional study	EAT-40
31	[86]	Ferrand and Brunet 2004	France	Female sex = 0%; mean age 21.8 years; mean BMI NR Kg/m^2^	Cross-sectional study	EAT-26
32	[87]	Filaire et al., 2011	France	Female sex = 50%; mean age 19.5 years; mean BMI NR Kg/m^2^	Cross-sectional study	EAT-26
33	[88]	Flatt et al., 2021	Multiple	Female sex = 92%; mean age 22 years; mean BMI NR Kg/m^2^	Cross-sectional study	RD
34	[22]	Fortes et al., 2014	Brazil	Female sex = 20%; mean age 22 years; mean BMI NR Kg/m^2^	Cross-sectional study	EAT-26
35	[20]	Ghazzawi et al., 2022	Jordan	Female sex = 41%; mean age 31 years; mean BMI 25 kg/m^2^	Cross-sectional study	EAT-26
36	[89]	Gibson et al., 2019	New Zealand	Female sex = 0%; mean age 22 years; mean BMI NR Kg/m^2^	Cross-sectional study	EDI-3
37	[1]	Giel et al., 2016	German	Female sex = 44%; mean age 16.3 years; mean BMI NR Kg/m^2^	Cross-sectional study	SCOFF
38	[90]	Glotz et al., a 2013	Brazil	Female sex = 0%; mean age 22 years; mean BMI NR Kg/m^2^	Cross-sectional study	M1 = EAT-26 M2 = BITE
39	[91]	Godoy-Izquierdo and Díaz 2021	Spain	Female sex = 100%; mean age 20.9 years; mean BMI 23.1 kg/m^2^	Cross-sectional study	EAT-26
40	[92]	Gouttebarge and Kerkhoffs, a 2017	Multiple	Female sex = 0%; mean age 22 years; mean BMI NR Kg/m^2^	Cohort study	RD
41	[93]	Gouttebarge et al., 2017	Multiple	Female sex = 64%; mean age 27.3 years; mean BMI NR Kg/m^2^	Cross-sectional study	RD
42	[94]	Gouttebarge et al., 2017	Multiple	Female sex = 50%; mean age 33 years; mean BMI NR Kg/m^2^	Cohort study	RD
43	[95]	Greenleaf et al., 2009	USA	Female sex = 100%; mean age 20 years; mean BMI 23 kg/m^2^	Cross-sectional study	QEDD
44	[53]	Gullivera et al., 2015	Australia	Female sex = 53%; mean age 24.9 years; mean BMI NR Kg/m^2^	Cross-sectional study	SCOFF
45	[96]	Hauck et al., 2020	German	Female sex = 56.4%; mean age 36.44 years; mean BMI 22.83 kg/m^2^	Cross-sectional study	EDDS
46	[97]	Hoch et al., 2011	USA	Female sex = 100%; mean age 23.2 years; mean BMI 19 kg/m^2^	Cohort study	EDEQ
47	[98]	Hoch et al., 2009	USA	Female sex = 100%; mean age 16.5 years; mean BMI 21.6 kg/m^2^	Cross-sectional study	EAT-26
48	[99]	Hopkinson and Lock, a 2004	USA	Female sex = 48%; mean age 22 years; mean BMI NR Kg/m^2^	Cross-sectional study	M1 = EAT-26 M2 = EDEQ
49	[100]	Hulley and Hill 2001	UK	Female sex = 100%; mean age 28.5 years; mean BMI 21.05 kg/m^2^	Cross-sectional study	EDEQ
50	[101]	Janout and Janoutová c 2004	Czech Republic	Female sex = 100%; mean age 22 years; mean BMI 18 kg/m^2^	Cross-sectional study	EAT-26
51	[102]	Joubert et al., 2022	USA	Female sex = 100%; mean age 22 years; mean BMI NR Kg/m^2^	Cross-sectional study	RD
52	[103]	Joubert et al., 2020	Multiple	Female sex = 23.1%; mean age 32.5 years; mean BMI 22.4 kg/m^2^	Cross-sectional study	EAT-26
53	[104]	Junge and Hauschild 2023	German	Female sex = 55.8%; mean age NR years; mean BMI NR Kg/m^2^	Cross-sectional study	EDE-QS
54	[105]	Kampouri et al., 2019	Greece	Female sex = 100%; mean age 23.66 years; mean BMI 22.07 kg/m^2^	Cross-sectional study	EDEQ
55	[106]	Karlson et al., 2001	USA	Female sex = 100%; mean age 19.89 years; mean BMI 20.21 kg/m^2^	Cross-sectional study	EDEQ
56	[107]	Karlsson et al., 2023	Sweden	Female sex = 100%; mean age 32.4 years; mean BMI NR Kg/m^2^	Cross-sectional study	EDEQ
57	[108]	Kennedy et al., 2017	USA	Female sex = 52.11%; mean age 40.3 years; mean BMI 23.7 kg/m^2^	Cross-sectional study	EAT-26
58	[109]	Kristjánsdóttir et al., a 2019	Iceland	Female sex = 68.9%; mean age 24.8 years; mean BMI NR Kg/m^2^	Cross-sectional study	M1 = EDEQ M2 = BULIT-R
59	[110]	Lauder et al., 1999	USA	Female sex = 100%; mean age 22 years; mean BMI NR Kg/m^2^	Cross-sectional study	EDI
60	[111]	Lauder et al., 1999	USA	Female sex = 100%; mean age 27.5 years; mean BMI 23.5 kg/m^2^	Cross-sectional study	EDI
61	[56]	Lichtenstein et al., 2021	Denmark	Female sex = 51%; mean age 19.3 years; mean BMI 21.55 kg/m^2^	Cross-sectional study	SCOFF
62	[112]	Marshall and Harber 1996	Canada	Female sex = 100%; mean age 20.8 years; mean BMI 23.5 kg/m^2^	Cross-sectional study	EDI
63	[113]	Martínez Rodríguez et al., 2015	Spain	Female sex = 35%; mean age 20.9 years; mean BMI NR Kg/m^2^	Cross-sectional study	EAT-26
64	[114]	Martinovic et al., 2022	China	Female sex = 45%; mean age 24.2 years; mean BMI 24.2 kg/m^2^	Cross-sectional study	ORTO-15
65	[115]	McLester et al., 2014	Multiple	Female sex = 100%; mean age 22 years; mean BMI NR Kg/m^2^	Cross-sectional study	EDI-2
66	[116]	Meng et al., b 2020	China	Female sex = 100%; mean age 20 years; mean BMI 18.6 kg/m^2^	Cross-sectional study	EDI-3
67	[117]	Michaels et al., 2023	USA	Female sex = 92.9%; mean age NR years; mean BMI NR Kg/m^2^	Cross-sectional study	RD
68	[118]	Monthuy-Blanc et al., a 2010	France	Female sex = 100%; mean age 14.1 years; mean BMI 19.3 kg/m^2^	Cross-sectional study	EDI-3
69	[54]	Muros et al., b 2020	Spain	Female sex = 15.5%; mean age 34.54 years; mean BMI 22.85 kg/m^2^	Cross-sectional study	SCOFF
70	[43]	Neves et al., a 2017	Brazil	Female sex = 100%; mean age 13.1 years; mean BMI NR Kg/m^2^	Cohort study T1& T2 & T3	EAT-26
71	[119]	Nichols et al., 2006	Multiple	Female sex = 100%; mean age 15.7 years; mean BMI 21.8 kg/m^2^	Cross-sectional study	EDEQ
72	[120]	Nieves et al., 2016	USA	Female sex = 100%; mean age 18.4 years; mean BMI 22.8 kg/m^2^	Cohort study	EDI
73	[121]	O'Connell et al., 2024	Canada	Female sex = 66.1%; mean age 20.1 years; mean BMI NR Kg/m^2^	Cross-sectional study	DESA-6
74	[122]	O'Connor et al., 1995	Georgia	Female sex = 100%; mean age 19.8 years; mean BMI NR Kg/m^2^	Cross-sectional study	EDI-2
75	[42]	Okano et al., c 2004	Multiple	Female sex = 100%; mean age 22 years; mean BMI 20.8 kg/m^2^	Cross-sectional study	EAT-26
76	[123]	O'Leary et al., b 2023	UK	Female sex = 100%; mean age NR years; mean BMI NR Kg/m^2^	Cross-sectional study	M1 = BEDA-Q M2 = FAST
77	[124]	Pallotto et al., 2022	USA	Female sex = 100%; mean age 19.9 years; mean BMI 22.4 kg/m^2^	Cross-sectional study	EAT-26
78	[125]	Peklaj et al., 2022	Slovenia	Female sex = 51%; mean age 22 years; mean BMI 21.6 kg/m^2^	Cross-sectional study	RD
79	[126]	Pensgaard et al., 2021	Norway	Female sex = 40%; mean age 26.86 years; mean BMI NR Kg/m^2^	Cross-sectional study	EDEQ
80	[127]	Pernick et al., 2006	USA	Female sex = 100%; mean age 15.7 years; mean BMI NR Kg/m^2^	Cross-sectional study	EDEQ
81	[15]	Petisco-Rodríguez et al., a 2020	Spain	Female sex = 100%; mean age 17.29 years; mean BMI 20.59 kg/m^2^	Cross-sectional study	M1 = EAT-40 M2 = SCOFF
82	[128]	Petrie et al., 2009	USA	Female sex = 100%; mean age 19.7 years; mean BMI 22.6 kg/m^2^	Cross-sectional study	QEDD
83	[129]	Pettersen et al., c 2016	Norway	Female sex = 100%; mean age 22 years; mean BMI NR Kg/m^2^	Cross-sectional study	EDI-2
84	[130]	Poucher et al., d 2022	Canada	Female sex = 50%; mean age 22 years; mean BMI NR Kg/m^2^	Cohort study	EAT-26
85	[131]	Prather et al., 2016	USA	Female sex = 100%; mean age 16.4 years; mean BMI 20.8 kg/m^2^	Cross-sectional study	EAT-26
86	[132]	Pritchett et al., 2021	Multiple	Female sex = 50%; mean age 27 years; mean BMI NR Kg/m^2^	Cross-sectional study	EDEQ
87	[133]	Rauh et al., 2010	USA	Female sex = 100%; mean age 15.7 years; mean BMI 21.7 kg/m^2^	Cohort study	EDEQ
88	[134]	Ravaldi et al., b 2003	Italy	Female sex = 0%; mean age 19.6 years; mean BMI 22.1 kg/m^2^	Cross-sectional study	EDE-12
89	[135]	Ravi et al., 2021	Finland	Female sex = 100%; mean age 22 years; mean BMI NR Kg/m^2^	Cross-sectional study	RD
90	[136]	Reinking and Alexander, b 2005	USA	Female sex = 100%; mean age 19.8 years; mean BMI NR Kg/m^2^	Cross-sectional study	EDI-2
91	[137]	Reinking, 2006	USA	Female sex = 100%; mean age 19.3 years; mean BMI 22.9 kg/m^2^	Cohort study	FAST
92	[138]	Riebl et al., 2007	USA	Female sex = 0%; mean age 31.6 years; mean BMI NR Kg/m^2^	Cross-sectional study	EAT-26
93	[52]	Robbeson et al., b 2015	USA	Female sex = 100%; mean age 19 years; mean BMI 21.2 kg/m^2^	Cross-sectional study	TFEQ
94	[139]	Roberts and Kreipe 2003	USA	Female sex = 100%; mean age 14.9 years; mean BMI NR Kg/m^2^	Cross-sectional study	EAT-26
95	[55]	Rogers et al., 2021	Australia	Female sex = 100%; mean age 19 years; mean BMI NR Kg/m^2^	Cross-sectional study	SCOFF
96	[31]	Rosendahl et al., 2009	German	Female sex = 36.5%; mean age 15.7 years; mean BMI NR Kg/m^2^	Cross-sectional study	EAT-26
97	[140]	Rousselet et al., 2017	France	Female sex = 37.3%; mean age 16.8 years; mean BMI NR Kg/m^2^	Cross-sectional study	EDI
98	[141]	Rouveix et al., 2007	France	Female sex = 50%; mean age 22 years; mean BMI 21 kg/m^2^	Cross-sectional study	EAT-26
99	[142]	Schtscherbyna et al., a 2009	Brazil	Female sex = 100%; mean age 14.6 years; mean BMI NR Kg/m^2^	Cross-sectional study	M1 = EAT-26 M2 = BITE M3 = BSQ
100	[143]	Sharps et al., a 2022	UK	Female sex = 100%; mean age 22 years; mean BMI NR Kg/m^2^	Cross-sectional study	M1 = FAST
101	[144]	Smith et al., 2020	Multiple	Female sex = 26.5%; mean age 20 years; mean BMI NR Kg/m^2^	Cross-sectional study	EAT-26
102	[145]	Sophia et al., 2022	USA	Female sex = 100%; mean age 30.8 years; mean BMI 19.41 kg/m^2^	Cross-sectional study	LEAF-Q
103	[146]	Staal et al., 2018	Denmark	Female sex = 50%; mean age 24.8 years; mean BMI 20.2 kg/m^2^	Cross-sectional study	EDI-3
104	[147]	Stackeov et al., 2023	Prague, Czechia	Female sex = 79%; mean age NR years; mean BMI NR Kg/m^2^	Pilot study	EAT-26
105	[148]	Sundgot-Borgen 1993	Norway	Female sex = 100%; mean age 20.3 years; mean BMI 21.9 kg/m^2^	Cross-sectional study	EDI
106	[149]	Sundgot-Borgen 1994	Norway	Female sex = 100%; mean age 20 years; mean BMI 21 kg/m^2^	Cross-sectional study	EDI
107	[150]	Sundgot-Borgen and Torstveit 2004	Norway	Female sex = 93%; mean age 22.3 years; mean BMI 22.6 kg/m^2^	Cross-sectional study	EDI
108	[151]	Sundgot‐Borgen et al., 2003	Norway	Female sex = 100%; mean age 21.4 years; mean BMI NR Kg/m^2^	Cross-sectional study	EDI
109	[152]	Syed et al., 2022	Pakistan	Female sex = 100%; mean age 23.57 years; mean BMI 21.97 kg/m^2^	Cross-sectional study	EAT-26
110	[153]	Teixidor-Batlle et al., 2021	Spain	Female sex = 51.08%; mean age 16.7 years; mean BMI 21.25 kg/m^2^	Cross-sectional study	EAT-26
111	[154]	Tenforde et al., 2022	Multiple	Female sex = 40.7%; mean age 13.2 years; mean BMI 19.2 kg/m^2^	Cross-sectional study	EDEQ
112	[155]	Terry et al., 1999	UK	Female sex = 43%; mean age 23.9 years; mean BMI NR Kg/m^2^	Cross-sectional study	EAT-26
113	[156]	Thein-Nissenbaum et al., 2011	USA	Female sex = 100%; mean age 15.4 years; mean BMI 21.1 kg/m^2^	Cohort study	EDEQ
114	[157]	Thein-Nissenbaum et al., 2014	USA	Female sex = 100%; mean age 15.3 years; mean BMI 21.1 kg/m^2^	Cohort study	EDEQ
115	[158]	Thiel et al., 1993	German	Female sex = 0%; mean age 21.1 years; mean BMI 21.05 kg/m^2^	Cross-sectional study	EDI
116	[159]	Thompson 2007	USA	Female sex = 19.6%; mean age 20.5 years; mean BMI NR Kg/m^2^	Cross-sectional study	RD
117	[160]	Thompsonnet al., a 2017	USA	Female sex = 100%; mean age 19.24 years; mean BMI 22.55 kg/m^2^	Cohort study	QEDD
118	[161]	Torres-McGehee et al., 2009	USA	Female sex = 100%; mean age 19.2 years; mean BMI 22.5 kg/m^2^	Cross-sectional study	EAT-26
119	[162]	Torres-McGehee et al., 2011	USA	Female sex = 100%; mean age 19.88 years; mean BMI 22.6 kg/m^2^	Cross-sectional study	EAT-26
120	[163]	Torres-McGehee et al., 2023	Columbia	Female sex = 69.3%; mean age 19.8 years; mean BMI 22.6 kg/m^2^	Cross-sectional study	EAT-26
121	[164]	Torstveit and Sundgot-Borgen 2005	Norway	Female sex = 100%; mean age 21.3 years; mean BMI 21.6 kg/m^2^	Cross-sectional study	EDI
122	[165]	Torstveit et al., 2008	Norway	Female sex = 100%; mean age 22.2 years; mean BMI 21.7 kg/m^2^	Cross-sectional study	EDI
123	[166]	Uriegas et al., a 2021	USA	Female sex = 69%; mean age 19.6 years; mean BMI 23.6 kg/m^2^	Cross-sectional study	M1 = EAT-26 M2 = ORTO-15
124	[167]	Uriegas et al., 2023	Columbia	Female sex = 69.6%; mean age 19.8 years; mean BMI 22.5 kg/m^2^	Cross-sectional study	EAT-26
125	[168]	Vardar et al., 2005	Turkey	Female sex = 100%; mean age 22 years; mean BMI NR Kg/m^2^	Cross-sectional study	EAT-40
126	[169]	Vardar et al., 2007	Turkey	Female sex = 100%; mean age 19 years; mean BMI 20 kg/m^2^	Cross-sectional study	EAT-40
127	[170]	Walberg and Johnston 1991	USA	Female sex = 100%; mean age 22 years; mean BMI NR Kg/m^2^	Cross-sectional study	EDI
128	[171]	Waryasz et al., 2020	USA	Female sex = 100%; mean age 26.9 years; mean BMI NR Kg/m^2^	Cross-sectional study	RD
129	[172]	Wheeler et al., 1986	Canada	Female sex = 50%; mean age 33.6 years; mean BMI NR Kg/m^2^	Cross-sectional study	EAT-26
130	[29]	Whitehead et al., d 2020	Australia	Female sex = 100%; mean age 32.9 years; mean BMI 22.9 kg/m^2^	Cross-sectional study	M1 = TFEQ M2 = EDI
131	[173]	Wollenberg et al., 2015	USA	Female sex = 100%; mean age 19.5 years; mean BMI 22.1 kg/m^2^	Cross-sectional study	EAT-26
132	[174]	Wu et al., 2022	Multiple	Female sex = 40.6%; mean age 13.2 years; mean BMI 19.1 kg/m^2^	Cross-sectional study	EDEQ

*ANIS* Anorexia Nervosa Inventory for Self-Rating, *BEDS-7* Binge Eating Disorder Screener-7, *DEBQ* Dutch Eating Behavior Questionnaire, *EAT-26* Eating Attitudes Test-26, *EAT-40* Eating Attitudes Test-40, *EDDS* The Eating Disorder Diagnostic Scale, *EDE-Q* Eating Disorder Examination- Questionnaire, *EDI* Eating Disorder Inventory-I/II, *FEDS* feeding and eating disorders, *ORTO-11* ORTO-11, *ORTO-15* ORTO-15, *QEDD* Questionnaire for Eating Disorder Diagnoses, *SCOFF* Sick, Control, One Stone, Fat, Food, *SD* Self-developed, *TFEQ* The Three-Factor Eating Questionnaire, *WCS* The Weight Concern Scale

### Data visualization and analysis (synthesis method)

With the understanding that the real effects will change over time, a traditional meta-analysis utilizing the random-effects approach was applied. We estimated and corrected for the between-study variation in effects using the DerSimonian-Laird technique and the generic inverse contrast technique with logit transformed (PLO) ratios. The random effect model was adopted (*e.g.*, EAT and SCOFF; Table 1) since various study sets assess distinct, conceptually connected consequences using various metrics. Ninety-five percent confidence intervals, and pooled prevalence are presented for each study.

A forest plot was used to present the data. Forest plots have the limitation of only offering confidence intervals at significant levels, *i.e.*, p < 0.05. Drapery plots and p-curve analysis were also used since confidence intervals are needed to ascertain whether results are significant and replicable. The p-value function is represented in the drapery plot by curves which stand for the forecast value from one study to the next, as well as for the combined meta-analytic values.

We employed the *I*^2^ statistic to estimate between-study variability; a result between 75 and 100% indicates a greater standard of heterogeneity. Additionally, we assessed heterogeneity using tau^2^ (τ^2^), tau (τ), as well as Cochran's Q statistics. The square root for the following is the H statistic: Cochran's χ2 heterogeneity statistic divided by the level of freedom. We utilized a standard Galbraith radial plot to illustrate heterogeneity, where the opposite of standard errors (horizontal axis) is shown in relation to the actual effect magnitude or results normalized with their corresponding standard errors (vertical axis). An arc displays matching effect sizes or outcomes just on the right side of an entire Galbraith plot.

Outlier inclusion could reduce the reliability and validity of meta-analysis. Studies are characterized as outliers whenever their confidence intervals do not match those of the pooled effects and sensitivity analysis can be used to address these studies. To avoid having an excessive amount of influence from any given study, we used a Jackknife sensitivity analysis to exclude studies one at a time. In this analysis, the primary meta-analysis is repeated one additional time for every study analyzed, with a different study being discarded every time.

Publication bias can occur if, for instance, studies with positive results are published more often than studies with negative results. As a first visual, funnel plots were used to examine publication bias. The Doi plot replaces the traditional scatter plot (funnel plot) of the ratio of accuracy to efficiency with a plot of the ratio of the normal quantile (z-score) to efficiency [37]. If there is asymmetry, either the two ends of the graph are unequally offset from the midpoint, or one end has more trials than the other. If there is no asymmetry, a line drawn perpendicular to the x-axis from the top of the Doi graph is expected to divide the graph into two areas of equal area; the LFK index quantifies the difference between the two areas as the difference between their respective areas under the graph and the number of studies included at each edge; an LFK index closer to 0, the more symmetrical the Doi graph; LFK index values outside the range between -1 and + 1 are considered consistent with asymmetry (*i.e.,* publication bias) [38]. The trim and fill method was applied (if needed) to develop modified point estimates. Peters' correlations and Egger’s regression also were employed as additional benchmarks for a more thorough analysis of publication bias.

Subgroup meta-analyses were utilized to look into varied results and respond to particular questions about various study characteristics or populations. Subgroup analyses were performed on categorical variables including participants’ nationality, culture (Western versus Eastern), and frequently included clinical characteristics. Sports energy system and sports category subgroup meta-analysis was performed to examine differences between aerobic, anaerobic, and mixed sports. Aerobic sports are defined as sports that require prolonged aerobic metabolism. This includes long-distance running, cycling, swimming, rowing, and cross-country skiing [39]. Anaerobic sports are defined as sports relying on anaerobic metabolism and involving short bursts of high-intensity activity such as sprinting, weightlifting, and gymnastics [39]. Mixed sports are defined as sports that involve both anaerobic and aerobic energy systems, *e.g.*, soccer, basketball, tennis, volleyball, lacrosse, and hockey [39].

Any subgroup of four papers or more was addressed by the subgroup meta-analyses, and all findings were represented by graphical representation utilizing forest plots.

In essence, meta-regressions are essentially regression models where the outcomes of one or more explanatory factors have been used to forecast the amount of variance. Meta-regressions were conducted to examine associations between sample characteristics and SRDE prevalence estimates [40]. When an explanatory variable is raised by one unit in a meta-regression study, the regression coefficient shows how the output variable changes [40]. R^2^ was used to calculate the effect size in statistically significant meta-regression models, where 1–8%, 9–24%, and ≥ 25% of the variance corresponded to small, medium, and major effect size [40].

All data were analyzed using statistical computer software termed R. All classic meta-analyses were carried out using the packages "meta" and "metafor”. Risk-of-bias plotlines were produced for use in evaluating quality, using the package "robvis". An overview plot (weighted) was developed for all studies to indicate the amount of data contained within every judgment for each topic. A traffic light plot shows the total risk as well as the bias risk for each domain.

## Results

### Descriptive results

The literature search, ending 7th January 2024, yielded 4178 relevant studies. One hundred and thirty-two unique studies involving one hundred and seventy-seven studies (due to multiple screening tools or multiple data collection times) with a total sample size of 70,950 participants (K = 177 data points; N of participants = 70,950) met our inclusion and exclusion criteria. The details of those studies are shown in Table 1. The PRISMA2020 process flow for research selection is shown in Fig. 1.

**Fig. 2:**
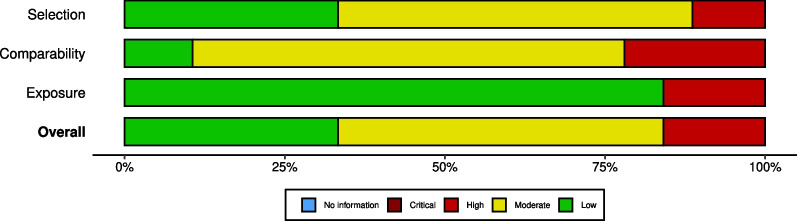
Summary risk of bias of the included studies

Twenty-seven countries were represented in our sample (Table 1), of which 92.1% were categorized as Western culture (33.3% were from the USA). Thirty percent of the studies addressed aerobic sports systems although most studies (59.9%) reported on sports that were mixed between aerobic and anaerobic exercise energy systems. Only 10.7% were purely anaerobic. Moreover, while 54.2% of the studies were of mixed indoor/outdoor sports, 26.5% were of purely outdoor sports. The EAT-26 was used in 31.1% of the studies (Table 2). A clear majority 88.1% of the studies were cross-sectional, while, in 11.9%, the design was longitudinal. None of the studies meeting the inclusion and exclusion criteria were conducted during the lockdown period of the COVID-19 pandemic. Figure 2 shows that approximately 80% of the studies were at low or moderate risk of bias. A detailed assessment of the risk of bias of each study is presented in Additional file 1.

**Table 2:** Random and common effects meta-analysis models of the prevalence of disordered eating in Athletes

Analysis	Descriptive		Random-effects meta-analysis	Common-effects meta-analysis	Visual Results	Heterogeneity						Moderators		Publication bias	
	K	N	Pooled results (95%CI)	Pooled results (95%CI)	Forest Plot	H	I^2^	τ^2^	τ	Q	p	Age	Sex	Egger's test	Rank test
All Data	177	70,957	19.23% [17.04; 21.62]	20.52% [20.15; 20.89]	Figure 3	6.17	97.4%	0.9	0.9	6699.62	0	NS	NS	NS	NS
By Country Turkey China Czech Republic German France UK Canada Spain Norway Australia Brazil Multi USA	3 3 4 5 7 7 8 10 10 11 12 17 59	580 466 172 2879 711 6558 857 6066 3506 1005 1258 10,888 27,017	30.4% [9.1; 65.6] 51.4% [40.7; 61.9] 28.3% [20.3; 37.9] 13.9% [8.3; 22.4] 29.0% [21.5; 37.9] 18.7% [12.3; 28.0] 7.7% [3.5; 16.4] 12.8% [8.01; 19.7] 22.6% [15.2; 32.3] 57.1% [36.0; 75.8] 20.8% [13.7; 30.4] 10.9% [7.0; 16.6] 17.0% [12.9; 22.0]	28.4% [24.4; 32.8] 48.7% [44.1; 53.2] 29.1% [22.7; 36.5] 16.3% [14.9; 17.8] 30.6% [27.2; 34.1] 14.0% [13.2; 14.9] 15.7%[12.6; 19.3] 20.0% [19.0; 21.1] 18.4% [17.1; 20.0] 39.1% [35.4; 42.9] 19.0% [16.7; 21.6] 11.2% [10.5; 12.0] 24.0% [23.2; 24.8]	Additional file 9	–	98.0% 75.7% 22.2% 95.6% 76.0% 95.6% 89.0% 97.6% 95.8% 96.2% 86.6% 97.2% 98.2%	1.7 0.1 0.05 0.4 0.2 0.3 1.2 0.6 0.6 2.0 0.6 1.0 1.5	1.3 0.3 0.2 0.6 0.4 0.5 1.1 0.8 0.8 1.4 0.8 1.0 1.2	101.8 8.2 3.9 91.0 25.0 137 63.7 377.1 215.9 265.0 82.3 577.8 3207.6	< 0.0001	–	–	–	–
By Culture Eastern Western	14 163	1980 68,977	29.1% [20.1; 40.2] 18.5% [16.3; 20.9]	33.6% [31.3; 35.9] 19.96% [19.95; 20.34]	Additional file 10	–	97.4% 94.5%	0. 9 0.8	0.9 0.9	6291.2 237.6	–	–	–	–	–
By sports energy system Anaerobic Aerobic Both	19 52 106	1828 16,287 52,842	37.9% [27.0; 50.2] 19.6% [15.2; 25.0] 17% [14.6; 19.4]	38.3% [35.8; 40.9] 25.5% [24.7; 26.4 18.1% [17.7; 18.5]	Additional file 11	–	93.9% 97.7% 97.2%	1.1 1.2 0.7	1.0 1.1 0.8	297.3 2187.52 3684.5	–	–	–	–	–
By sport category Swimming Martial arts Gymnastics Outdoor Mixed	3 11 20 47 96	234 507 1680 29,195 39,341	20.2% [8.6; 40.6] 26.3% [18.1; 36.4] 41.5% [30.4; 53.6] 15.4% [11.6; 20.2] 17.6% [15.2; 20.4]	25.2% [19.6; 31.7] 25.8% [21.8; 30.2] 40.9% [38.2; 43.6] 15.8% [16.2; 17.4] 21.3% [20.9; 21.8]	Additional file 12	–	88.3% 76.6% 92.9% 98.0% 97.4%	0.7 0.5 1.0 1.2 0.7	0.8 0.7 1.0 1.1 0.9	17.1 42.7 267.5 2154.3 3714.3	< 0.0001	–	–	–	–
By Measurement tool BITE TFEQ EAT–40 QEDD FAST EDI–2 EDI–3 SCOFF RD EDI EDEQ EAT–26	3 5 5 6 6 8 9 9 13 19 25 55	258 228 804 2336 4371 1034 968 6775 6398 4513 9145 27,055	12.2% [2.8; 40.3] 73.0% [60.1; 82.8] 18.6% [6.7; 42.1] 7.3% [2.9; 17.1] 22.0% [11.6; 37.7] 15.0% [10.5; 20.9] 34.0% [25.4; 43.9] 19.4% [13.9; 26.6] 14.8% [10.6; 20.2] 35.1% [27.2; 44.0] 15.1% [10.4; 21.3] 15.9% [12.4; 20.1]	19.5% [13.6; 27.1] 75.4% [69.1; 80.7] 23.9% [20.6; 27.5] 16.9% [15.0; 19.0] 18.0% [16.8; 19.3] 13.5% [11.5; 15.9] 34.9% [31.9; 38.1] 21.1% [20.1; 22.2] 13.6% [12.8; 14.6] 25.1% [23.7; 26.5] 19.1% [18.0; 20.2] 19.0% [18.4; 19.7]	Additional file 13	–	90.0% 65.7% 97.0% 97.4% 98.3% 71.5% 86.0% 97.3% 94.9% 95.6% 96.9% 97.4%	1.7 0.3 1.7 1.4 0.9 0.2 0.3 0.3 0.4 0.6 1.1 1.0	1.3 0.5 1.3 1.2 0.9 0.5 0.6 0.6 0.6 0.8 1.0 1.0	20.0 11.7 135.0 194.8 292.5 24.5 57.0 292.5 233.8 408.3 778.7 2095.6	–	–	–	–	–
By Design Cohort study Cross–sectional	21 156	3419 67,538	22.2% [18.1; 27.0] 19.1% [16.8; 21.7]	25.7% [24.1; 27.3] 20.1% [19.8; 20.7]	Additional file 14	–	86.2% 97.6%	0.3 0.9	0.5 1.0	145.2 6504	–	–	–	–	–

K included studies numbers,

N included samples numbers

I^2^ Statistic refereed to the percentage of variation across samples due to heterogeneity rather than chance

τ^2^ Describe the extent of variation among the effects observed in different samples (between-sample variance)

H Describe confidence intervals of heterogeneity

^d^Significant differences between samples in meta-analysis

^e^Detects publication bias in meta-analysis

^f^Represent the correlation between effect sizes and sample variation

### Meta-analysis of the prevalence of SRDE

**Fig. 3:**
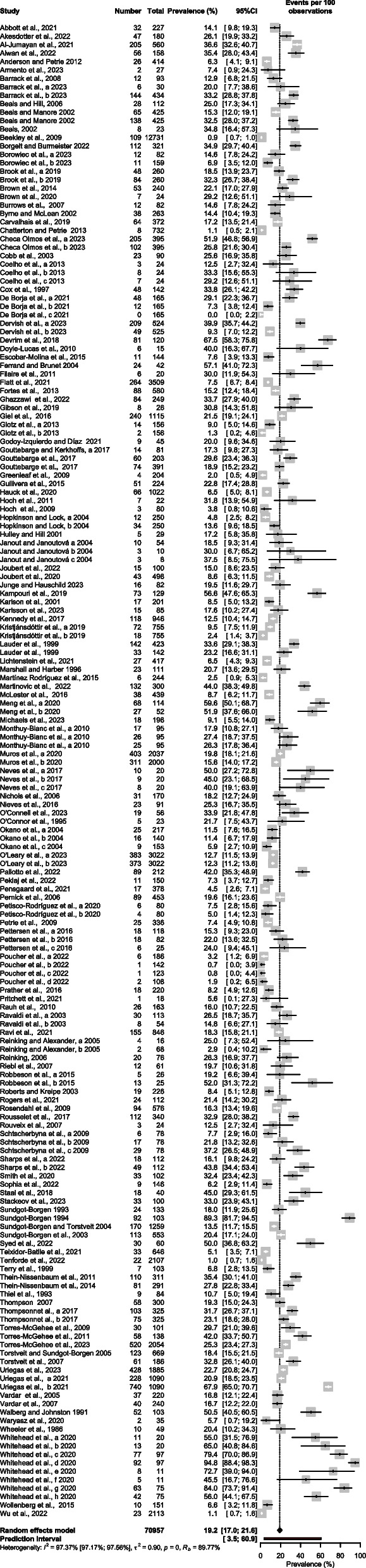
Meta-analysis of disordered eating in athletes

**Fig. 4:**
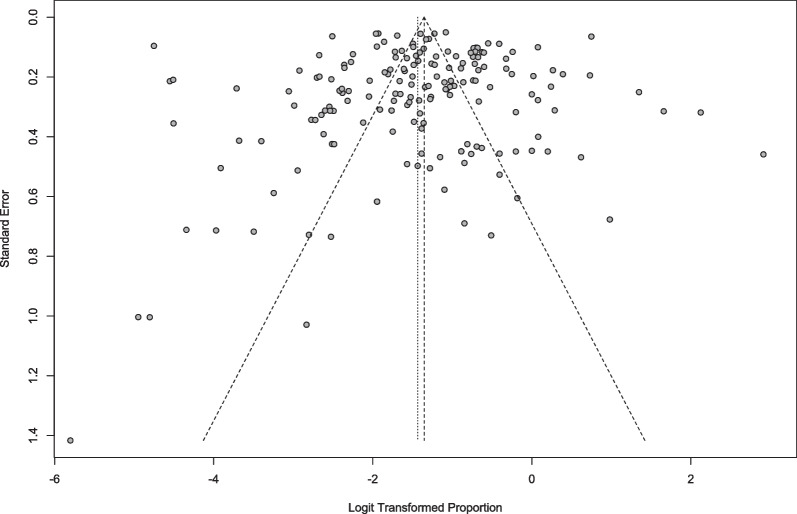
Funnel plot of disordered eating in athletes

Meta-analysis results and the raw prevalence data of each study are presented in Fig. 3. According to the random-effects meta-analysis, the SRDE prevalence among athletes (*K* = 177, *N* = 70,957) was (95% CI) = 19.23% (17.04%; 21.62%), *I*^2^ = (95% CI) = 97.4% (97.2%-97.6%),*τ* (95% CI) = 0.9481 (0.9374; 1.2182), τ2 = 0.8990 (0.8787–1.4840), *H* (95% CI) = 6.17 (5.95%-6.40%), *p*-value Cochran's *Q* = 0.00. Visual inspection of the funnel plot (Fig. 4), radial plot (Additional file 2), DOI plot (Additional file 3) as well as non-significant (at 0.05) Egger's regression and Peter's tests, indicated that the likelihood of publication bias was low. Additional file 4 represents the Drapery plot of DE in athletes; the drapery plot displays all primary study P-value functions in one graph, along with a P-value curve for each pooled estimate and a shaded prediction region. In contrast to the forest plot, the drapery plot displays results for any possible confidence level, not just one arbitrary confidence level. The results from our Drapery plot confirm that the results of the original/primary studies included are replicable. Additional file 5 presents possible outliers. The effect of outliers was minimal and required no accommodation.

### Meta-regression and subgroup meta-analyses

Table 2 presents the analyses of subgroups, with the stipulation that there are at least three studies in a subgroup.

### Age, BMI, and sex

Meta-regression analyses (Additional file 6, Additional file 7, and Additional file 8, respectively) demonstrate that age, BMI, and sex were statistically significant predictors/correlates, all p < 0.01. The effect sizes were very small: age R^2^ =  ~ 0.00%; BMI R^2^ =  ~ 4.5%; and sex R^2^ = 0.00%.

### Country and culture

Additional file 9 and Additional file 10 show the weighted prevalence levels of SRDE as a function of the country and culture in which the data were collected. These varied substantially, and, as noted above, for many countries the number of studies meeting the inclusion and exclusion criteria was low. Australia (*K* = 11, *N* = 1,005) at 57.1% (36.0%- 75.8%) had the greatest prevalence of SRDE among athletes, followed by Greece (*k* = 1, *N* = 129) at 56.6% (47.9%-64.9%), followed by China (*k* = 3, *N* = 466) at 51.38% (40.7%-61.9%), then Pakistan (*k* = 1, *N* = 60) at 50% (37.6%-62.4%). The United States had the highest number of included studies (*k* = 59, *N* = 27,017). Nine countries had only one study and the results were distinctly divergent; Sweden reported SRDE of ~ 22.5%, Jordan 33.7%, Saudi Arabia 36.6%, New Zealand 30.8%, Portugal 17.2%, and Slovenia 7.3%.

As a group, Western countries (*k* = 163, *N* = 68,977) had a slightly lower prevalence of SRDE among athletes at 18.5% (16.3%-20.9%) than Eastern countries (*k* = 14, *N* = 1,980) at 29.1% (20.1%-40.2%), with statistically significant differences between East and West (*p* = 0.02).

### Sports energy system and sports category

Additional file 11 shows the weighted prevalence levels of SRDE according to the energy system (aerobic vs anaerobic) of the sport. There were large variations. Most studies reported a mix of energy systems (*k* = 106, *N* = 52,842) with 17% (14.6%-19.4%). However, aerobic energy system sports were nearly three times more often reported on as anaerobic ones, (*k* = 52, *N* = 16,287) versus (*k* = 19, *N* = 1,828) respectively. Anaerobic sports had the highest prevalence rate of SRDE at 37.9% (27%-50.2%). Aerobic sports came next at 19.6% (15.2%-25%), while mixed sports showed the lowest prevalence rate of SRDE at 17% (14.6%-19.4%).

Results according to the sport were very similar, as shown in Additional file 12, which illustrates the fact that mixed sports were most commonly studied and swimming was the sport least studied, (*k* = 96, *N* = 39,341) versus (*k* = 3, *N* = 234). The martial arts (*k* = 11, *N* = 507) versus (*k* = 47, *N* = 29,195) for outdoor sports. Gymnastics had the highest prevalence rate of SRDE at 41.5% (30.4%-53.6%). Martial arts had the next highest prevalence rate of SRDE at 26.3% (18.1%-36.4%), while outdoor sports had the lowest prevalence rate of SRDE at 15.6% (11.6%-20.2%).

### Measures of SRDE

Meta-analysis showed significant heterogeneity amongst the measurement tools used in the studies (Table 2, Additional file 13) *I*^2^ = 97.4%, *τ*^2^ = 0.8990, *p* = 0.00. Of all 22 measures, the TFEQ = Three-Factor Eating Questionnaire (*k* = 5, *N* = 228) produced the highest prevalence at 73% (60.1; 82.8%), while the QEDD or Questionnaire for Eating Disorder Diagnoses (*k* = 6, *N* = 2,336) yielded the lowest prevalence at 7.3% (2.9%-17.1%). The main measure used, the EAT-26, yielded a prevalence of 15.9% (12.4%; 20.1%), (*k* = 55, *N* = 27,055).

Seven tools were used only once (*k* = 1); the Leisuretime Exercise and Anorexia Nervosa Questionnaire (LEAF-Q) (*N* = 146), the Bulimia Test Revised (BULIT-R) (*N* = 755), the Eating Disorder Diagnostic Scale (EDDS) (*N* = 1022), the Eating Disorder Screen for Primary Care (ESP) (*N* = 165), the Disordered Eating Screen for Athletes (DESA-6) (*N* = 56), the Structured Inventory for Anorexic, the Bulimic Syndromes (SIAB-S) (*N* = 82) and Female Athlete Screening Tool (*N* = 4371).

### Study design

Almost one-fifth of studies (*k* = 156) were cross-sectional involving (*N* = 67,538) participants. Only 21 studies were cohort studies, with 3,419 participants. The prevalence of SRDE in cohort studies was 22.2% (18.1%-27%), as shown in Additional file 14.

## Discussion

The purpose of this meta-analysis was to estimate the prevalence of DE in athletes, based on published studies that used validated screening measures. The search and selection process resulted in 177 studies, conducted in 27 countries (total *N* of participants = 70,957). This meta-analysis indicates an estimate of CI 95% = 19.23% (17.0%-21.6%) as the global prevalence of SRDE in athletes. This is higher than in the general population [20]. Given the physical and mental health consequences of DE and considering its status as a risk factor for clinically significant EDs, the prevalence rates are considered high [20].

The most frequently associated with SRDE was competing in gymnastics and sports divided along weight lines [20, 41–43]. With advance knowledge of risk, health care professionals are better equipped to provide early treatment when required [17, 44]. In general practice, this knowledge paired with an ongoing patient-provider trusting relationship, puts the physician in a unique position to diagnose and treat SRDE, thus preventing progression to an ED [23]. Knowledge about risk factors is also important for health promotion and targeted preventive measures. Results of this research are recommended reading for athletes as well as trainers, coaches, physicians, Olympic committees and administrators of athletic organizations [28, 45–47].

The prevalence estimate of 19% was, significantly associates with age, the proportion of females in the sample, and sample's mean BMI level. The prevalence of SRDE was higher in Eastern countries. Of interest to Arab countries, the prevalence of SRDE in athletes was only available in two Arabic countries: 36.6% among Saudi athletes [48] and, in Jordan 33.7% [20].

Since self-screening leads to early risk identification and effective prevention, it will be important to survey self-identified DE and examine its history and symptom patterns in more detail [49]. In a recent study of 249 Jordanian athletes aged over 18 years, Ghazzawi et al. [20] found an overall prevalence of SRDE of 33.7%, which is far higher than the mean estimate of 19.23% in our meta-analysis. In the Ghazzawi et al., 2022 study, outdoor sports (running, cycling, and walking), relevant to 8% of the participants, showed the lowest percentage of DE (3% of the 34%); the highest percentage (10% out of the 34%) was among gymnasts who accounted for 30% of all participants[20].

More studies, using established epidemiological methods (*e.g.*, representative sampling), are needed to clarify the prevalence and correlates of SRDE in athletes. In this regard, the published research to date has been limited to samples from only 27 countries, that is about 10% of the world's approximately 250 independent territorial entities. Notably absent in the literature that met our criteria are studies from Latin American countries (other than one from Brazil), and there is either zero or one sole study from several countries that have contributed to the general literature on DE, such as Sweden, New Zealand, Portugal, Slovenia and the Middle East countries (other than Saudi Arabia and Jordan).

Researchers seeking to understand the prevalence of DE as a multifaceted construct have many screening tools from which to choose (Table 1). Based on the substantial variability (heterogeneity) of the prevalence estimates in our meta-analysis, and in order to facilitate comparisons across studies from different countries while avoiding estimates that are almost certainly far too high or too low, we recommend the standardized use of the EAT-26 [50] plus the TFEQ [51].

The EAT-26 has been extensively validated across ages, sex groups, and cultural contexts as a screening tool with strong psychometric properties for identifying DE attitudes and behaviors [50]. The TFEQ thoroughly assesses different problematic eating patterns such as restrained-, and emotional- eating that may represent risk factors for the development of EDs [29, 52]. Together, these two instruments capture a comprehensive range of symptoms and eating behavior anomalies indicative of risk. The self-report format makes them possible to use for large population screenings where clinician interviews are impractical. Furthermore, both questionnaires have been translated and validated in various languages, enabling consistent implementation globally [29, 52]. Standardizing to widely-used instruments like the EAT-26 and TFEQ with robust psychometric properties that comprehensively assesses DE will help generate prevalence estimates across studies that are more homogeneous, allowing for meaningful cross-national comparisons and meta-analytic examination [29, 52].

The TFEQ, which was used in five data points [29, 52] included in this meta-analysis, is a valid and widely used measure of ED behaviors, and, therefore, it can add behavioral information to the screening items included in the EAT-26. If the EAT-26 is impractical due to its length, then we recommend substituting the 5-item SCOFF, which, according to Table 1, has thus far been used in only seven studies of SRDE in athletes [15, 53–56].

TFEQ has been previously used in studies samples that included athletes, its psychometric properties and validity have not yet been investigated specifically in athletic populations, representing an important direction for further research [51].

Furthermore, the hormonal and metabolic dysregulation of DE can negatively impact athletic performance in various ways. Decreased estrogen and testosterone levels hinder the building and maintenance of muscle mass [11, 57]. Reduced IGF-1 and growth hormone lead to impaired bone development, and increased fracture risk [11, 57, 58]. Electrolyte abnormalities from purging behaviors can disrupt cardiac function [57, 58]. Low energy availability alters substrate metabolism, making it difficult to meet energy demands during training and competition [11, 57]. Extreme weight loss and nutritional deficiencies can also impede recovery after exercise [11, 57, 58].

### Study strengths and limitations

To the best of our knowledge, this is the first meta-analysis of the prevalence of SRDE among athletes. The substantial number of studies and participants included adds strength to this review. Limitations include the reliance of many studies on convenience samples rather than representative samples and our inability to review articles published in languages other than English. The limited nature of the information about participants also ruled out statistical examination of potentially important moderating variables such as ethnicity, immigration status, sexual orientation, and family history.

An additional limitation of our analysis stems from the small number of studies conducted in Eastern countries, as well as our ability to examine differences among countries solely along the Western vs. Eastern dichotomy. The small sample of studies from Eastern nations limits conclusions about the seemingly high prevalence rates found in these settings. Furthermore, grouping diverse countries into binary Western and Eastern categories obscures important heterogeneity within regions in terms of specific sociocultural factors influencing DE. Future research should include more studies across a wider range of Eastern countries and examine country-level differences using more nuanced categorization systems. Relatedly, our study was limited by its focus on the gender binary, when DE prevalence among transgender and nonbinary populations warrants dedicated investigation.

To maximize the breadth of the literature captured, we did manually search the reference lists of included studies to help identify any relevant articles that our search may have missed. However, we acknowledge that it is possible we may have inadvertently excluded some studies by not including a wider range of activity-specific terms in the database searches. For future updates to this meta-analysis, we will consider broadening the search to include additional keywords related to different sports, as well as other physical activities such as dance, mountain climbing, boating, and others.

## Conclusion

The prevalence of SRDE in a meta-analysis of a very large sample of athletes from 27 countries was shown to be 19.23%, or one in every five athletes. DE is a health problem in itself, but it is also a strong risk factor for EDs. Our meta-analysis strongly suggests that, in the sports world, it affects females and males to an equal degree. While concentrating on sports injuries, public health has neglected the nutritional needs of athletes and the health consequences of dietary deficiencies in persons with high energy expenditure.

### Supplementary Information

**Additional file 1** Traffic light of the included studies.

**Additional file 2** Radial plot of disordered eating in athletes.

**Additional file 3** DOI plot of disordered eating in athletes.

**Additional file 4** Drapery plot of disordered eating in athletes.

**Additional file 5** Outlier analysis.

**Additional file 6** Meta-regression of disordered eating in athletes by Age.

**Additional file 7** Meta-regression of disordered eating in athletes by BMI.

**Additional file 8** Meta-regression of disordered eating in athletes by Sex (%Female Sex).

**Additional file 9** Subgroup meta-analysis by country.

**Additional file 10 **Subgroup meta-analysis by culture.

**Additional file 11** Subgroup meta-analysis by the sports energy system .

**Additional file 12** Subgroup meta-analysis by sports category.

**Additional file 13** Subgroup meta-analysis by disordered eating measurement tool.

**Additional file 14** Subgroup meta-analysis by study design.

## Data Availability

Data is available in Table 1.
