# Supplementary material for: A systematic review, meta-analysis, and meta-regression of the prevalence of self-reported disordered eating and associated factors among athletes worldwide
Source: J Eat Disord. 2024 Feb 7;12:24. doi: 10.1186/s40337-024-00982-5 (PMC10851573; doi:10.1186/s40337-024-00982-5)
Supplement: Supplementary file 1 — Additional file 1 Traffic light of the included studies. [file 40337_2024_982_MOESM1_ESM.pdf]

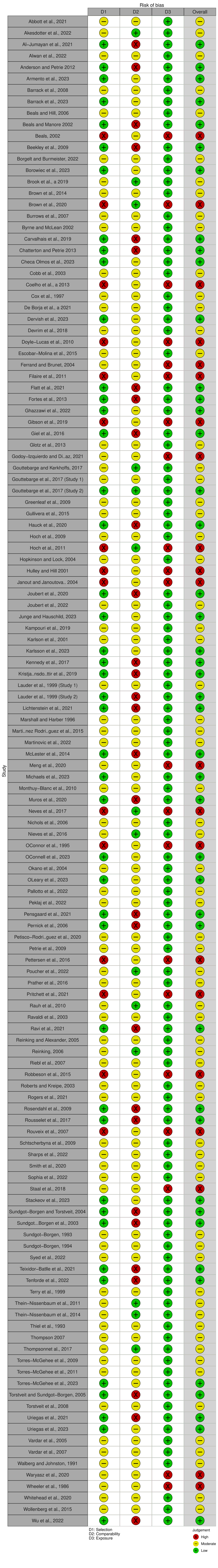
Risk of bias
D1
D2
D3
Overall
Study
Abbott et al., 2021
Akesdotter et al., 2022
Al–Jumayan et al., 2021
Alwan et al., 2022
Anderson and Petrie 2012
Armento et al., 2023
Barrack et al., 2008
Barrack et al., 2023
Beals and Hill, 2006
Beals and Manore 2002
Beals, 2002
Beekley et al., 2009
Borgelt and Burmeister, 2022
Borowiec et al., 2023
Brook et al., a 2019
Brown et al., 2014
Brown et al., 2020
Burrows et al., 2007
Byrne and McLean 2002
Carvalhais et al., 2019
Chatterton and Petrie 2013
Checa Olmos et al., 2023
Cobb et al., 2003
Coelho et al., a 2013
Cox et al., 1997
De Borja et al., a 2021
Dervish et al., 2023
Devrim et al., 2018
Doyle–Lucas et al., 2010
Escobar–Molina et al., 2015
Ferrand and Brunet, 2004
Filaire et al., 2011
Flatt et al., 2021
Fortes et al., 2013
Ghazzawi et al., 2022
Gibson et al., 2019
Giel et al., 2016
Glotz et al., 2013
Godoy–Izquierdo and Di..az, 2021
Gouttebarge and Kerkhoffs, 2017
Gouttebarge et al., 2017 (Study 1)
Gouttebarge et al., 2017 (Study 2)
Greenleaf et al., 2009
Gullivera et al., 2015
Hauck et al., 2020
Hoch et al., 2009
Hoch et al., 2011
Hopkinson and Lock, 2004
Hulley and Hill 2001
Janout and Janoutova.. 2004
Joubert et al., 2020
Joubert et al., 2022
Junge and Hauschild, 2023
Kampouri et al., 2019
Karlson et al., 2001
Karlsson et al., 2023
Kennedy et al., 2017
Kristja..nsdo..ttir et al., 2019
Lauder et al., 1999 (Study 1)
Lauder et al., 1999 (Study 2)
Lichtenstein et al., 2021
Marshall and Harber 1996
Marti..nez Rodri..guez et al., 2015
Martinovic et al., 2022
McLester et al., 2014
Meng et al., 2020
Michaels et al., 2023
Monthuy–Blanc et al., 2010
Muros et al., 2020
Neves et al., 2017
Nichols et al., 2006
Nieves et al., 2016
OConnor et al., 1995
OConnell et al., 2023
Okano et al., 2004
OLeary et al., 2023
Pallotto et al., 2022
Peklaj et al., 2022
Pensgaard et al., 2021
Pernick et al., 2006
Petisco–Rodri..guez et al., 2020
Petrie et al., 2009
Pettersen et al., 2016
Poucher et al., 2022
Prather et al., 2016
Pritchett et al., 2021
Rauh et al., 2010
Ravaldi et al., 2003
Ravi et al., 2021
Reinking and Alexander, 2005
Reinking, 2006
Riebl et al., 2007
Robbeson et al., 2015
Roberts and Kreipe, 2003
Rogers et al., 2021
Rosendahl et al., 2009
Rousselet et al., 2017
Rouveix et al., 2007
Schtscherbyna et al., 2009
Sharps et al., 2022
Smith et al., 2020
Sophia et al., 2022
Staal et al., 2018
Stackeov et al., 2023
Sundgot–Borgen and Torstveit, 2004
Sundgot...Borgen et al., 2003
Sundgot–Borgen, 1993
Sundgot–Borgen, 1994
Syed et al., 2022
Teixidor–Batlle et al., 2021
Tenforde et al., 2022
Terry et al., 1999
Thein–Nissenbaum et al., 2011
Thein–Nissenbaum et al., 2014
Thiel et al., 1993
Thompson 2007
Thompsonnet al., 2017
Torres–McGehee et al., 2009
Torres–McGehee et al., 2011
Torres–McGehee et al., 2023
Torstveit and Sundgot–Borgen, 2005
Torstveit et al., 2008
Uriegas et al., 2021
Uriegas et al., 2023
Vardar et al., 2005
Vardar et al., 2007
Walberg and Johnston, 1991
Waryasz et al., 2020
Wheeler et al., 1986
Whitehead et al., 2020
Wollenberg et al., 2015
Wu et al., 2022
D1: Selection
D2: Comparability
D3: Exposure
Judgement
High
Moderate
Low
