# Supplementary figures and images for: A systematic review, meta-analysis, and meta-regression of the prevalence of self-reported disordered eating and associated factors among athletes worldwide
Source: J Eat Disord. 2024 Feb 7;12:24. doi: 10.1186/s40337-024-00982-5 (PMC10851573; doi:10.1186/s40337-024-00982-5)

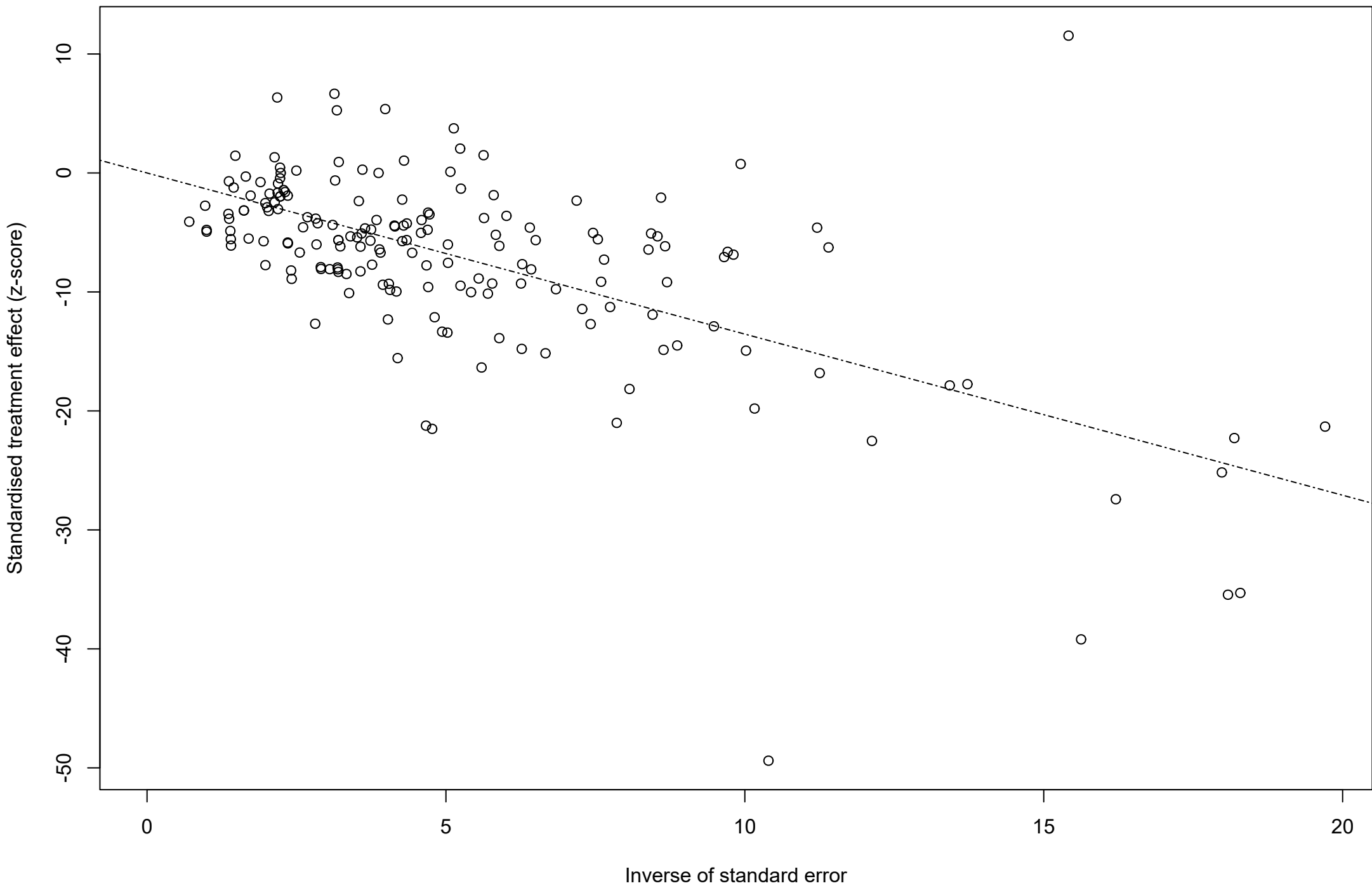

Supplement: Supplementary file 2 — Additional file 2 Radial plot of disordered eating in athletes. [file 40337_2024_982_MOESM2_ESM.pdf]

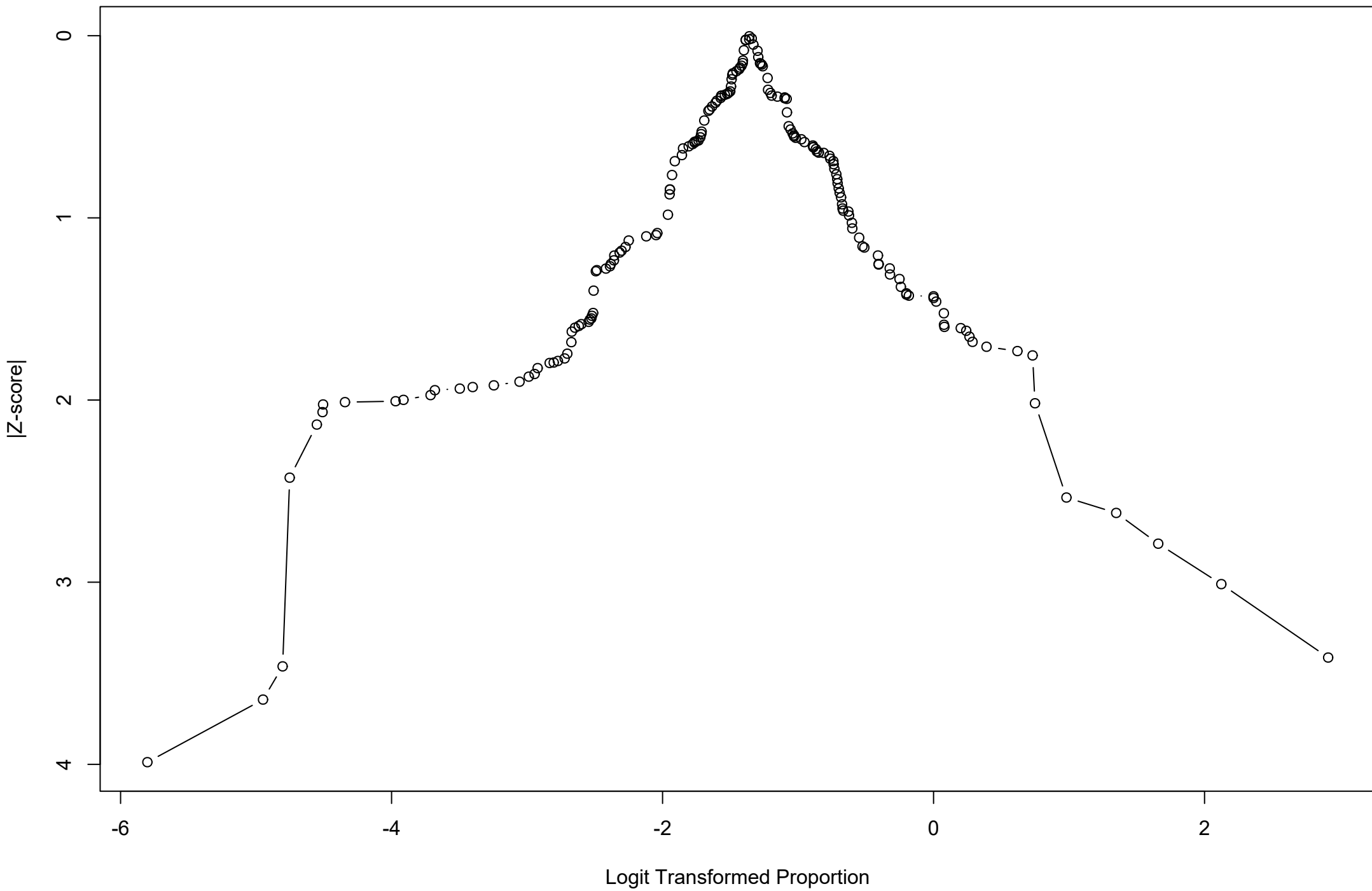

Supplement: Supplementary file 3 — Additional file 3 DOI plot of disordered eating in athletes. [file 40337_2024_982_MOESM3_ESM.pdf]

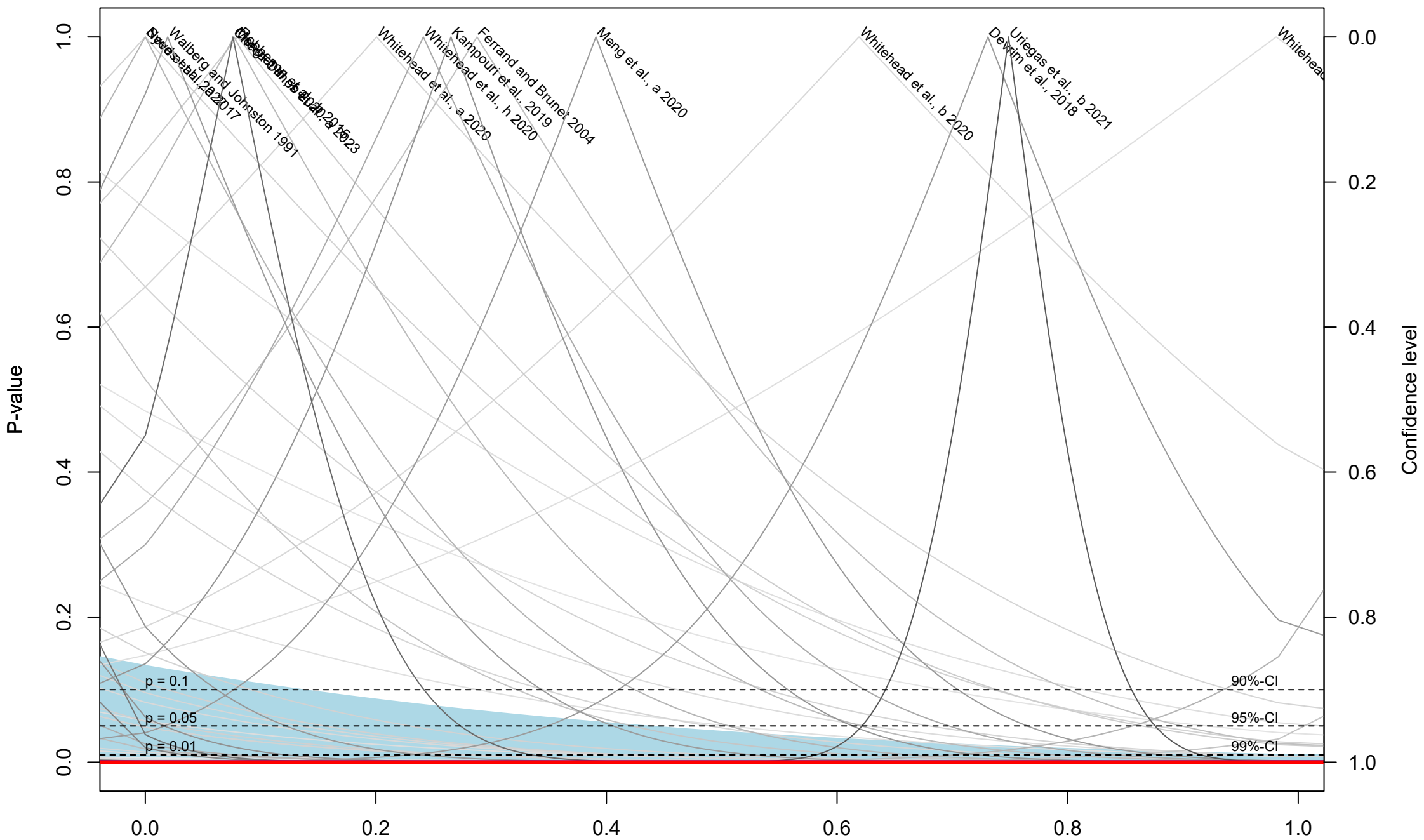

Supplement: Supplementary file 4 — Additional file 4 Drapery plot of disordered eating in athletes. [file 40337_2024_982_MOESM4_ESM.pdf]

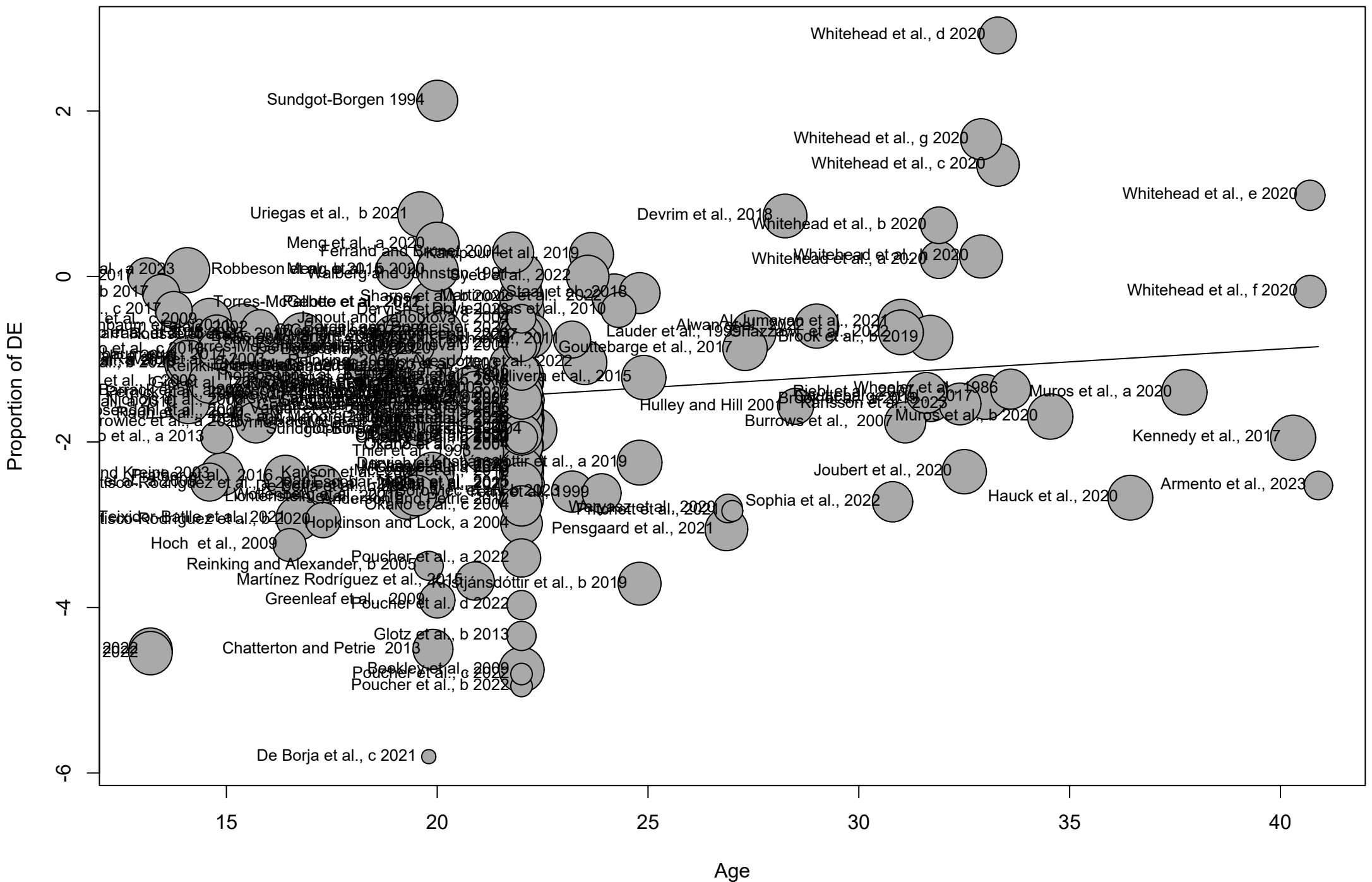

Supplement: Supplementary file 6 — Additional file 6 Meta-regression of disordered eating in athletes by Age. [file 40337_2024_982_MOESM6_ESM.pdf]

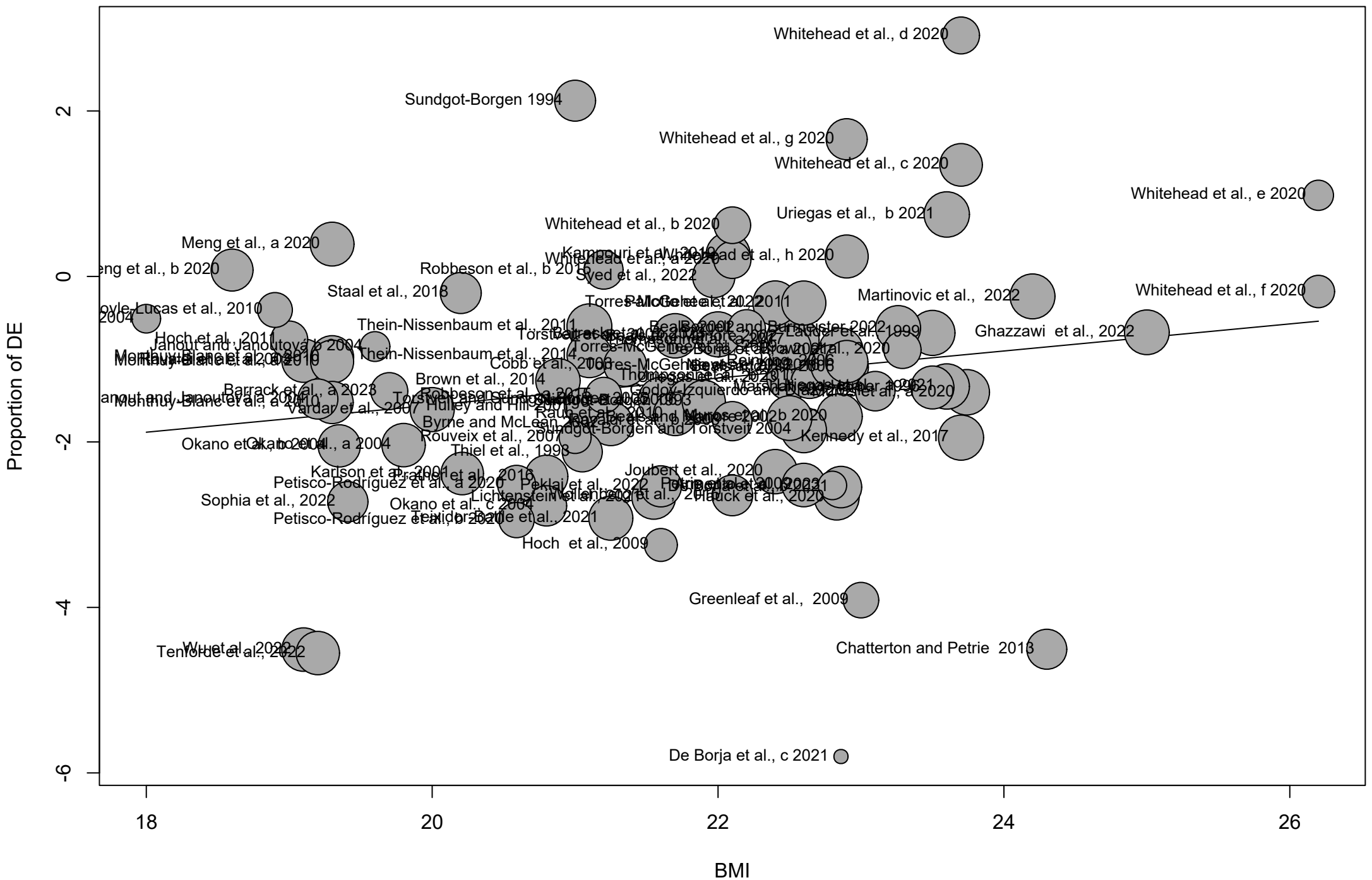

Supplement: Supplementary file 7 — Additional file 7 Meta-regression of disordered eating in athletes by BMI. [file 40337_2024_982_MOESM7_ESM.pdf]

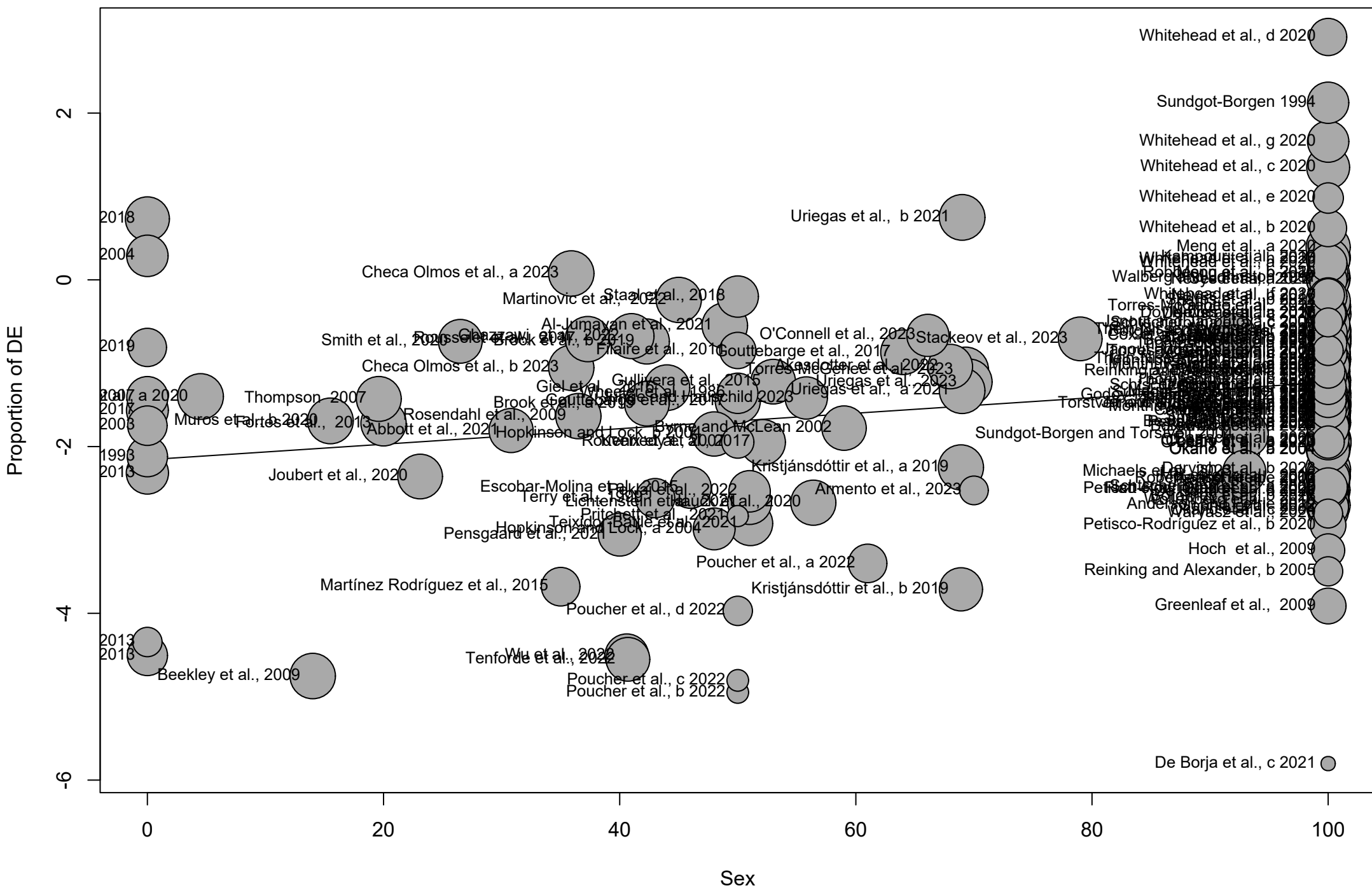

Supplement: Supplementary file 8 — Additional file 8 Meta-regression of disordered eating in athletes by Sex (%Female Sex). [file 40337_2024_982_MOESM8_ESM.pdf]

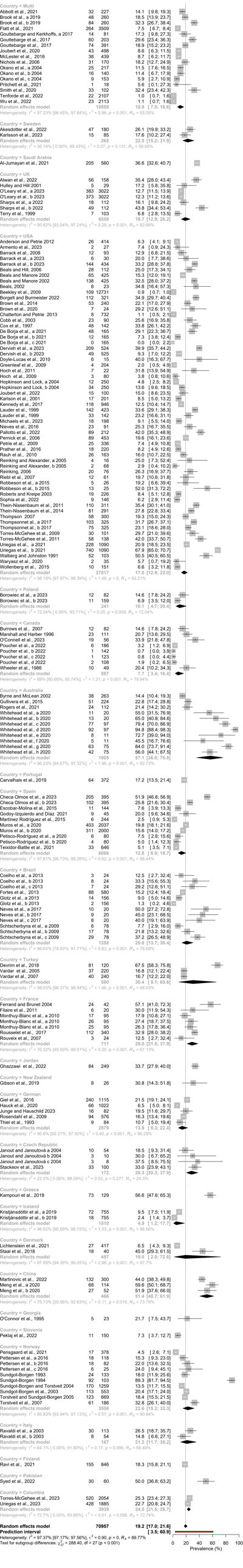

Supplement: Supplementary file 9 — Additional file 9 Subgroup meta-analysis by country. [file 40337_2024_982_MOESM9_ESM.pdf]

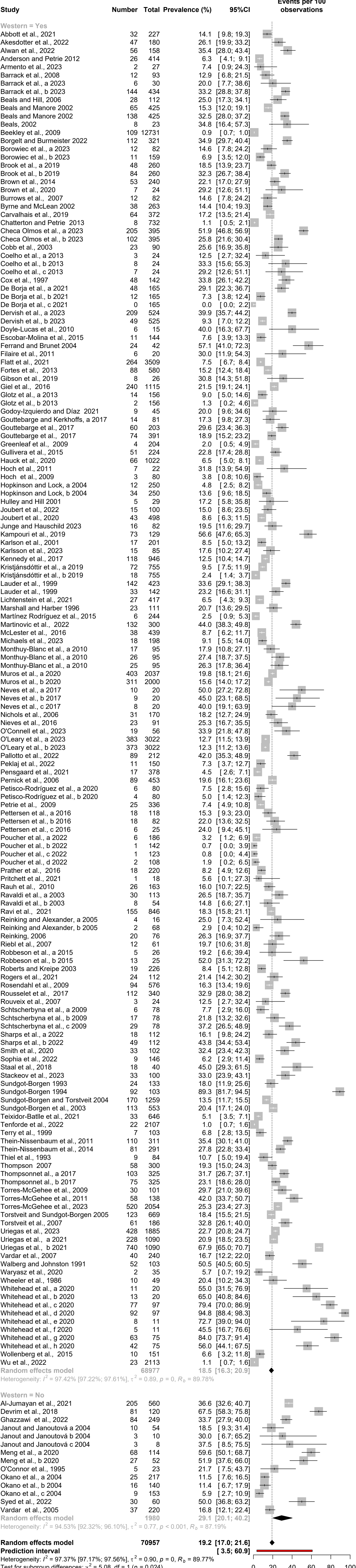

Supplement: Supplementary file 10 — Additional file 10 Subgroup meta-analysis by culture. [file 40337_2024_982_MOESM10_ESM.pdf]

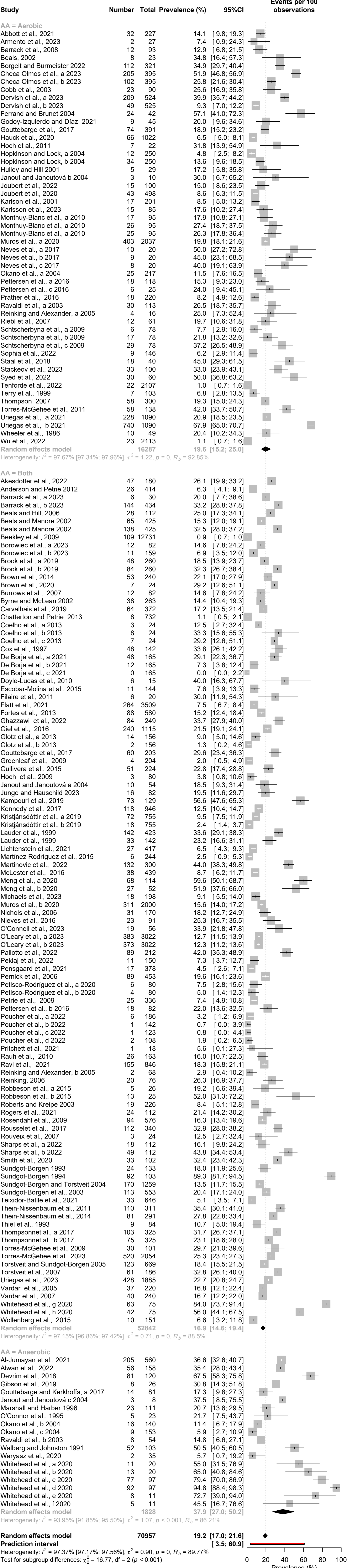

Supplement: Supplementary file 11 — Additional file 11 Subgroup meta-analysis by the sports energy system . [file 40337_2024_982_MOESM11_ESM.pdf]

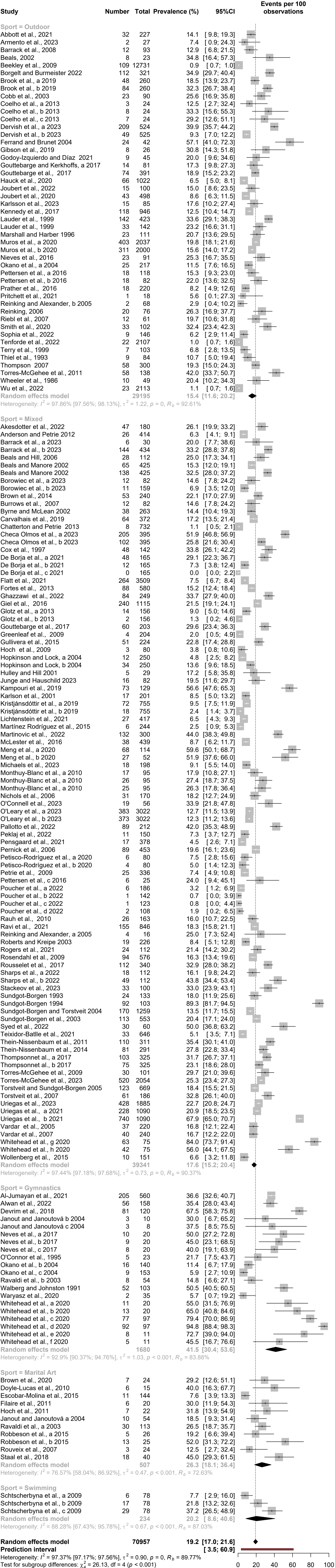

Supplement: Supplementary file 12 — Additional file 12 Subgroup meta-analysis by sports category. [file 40337_2024_982_MOESM12_ESM.pdf]

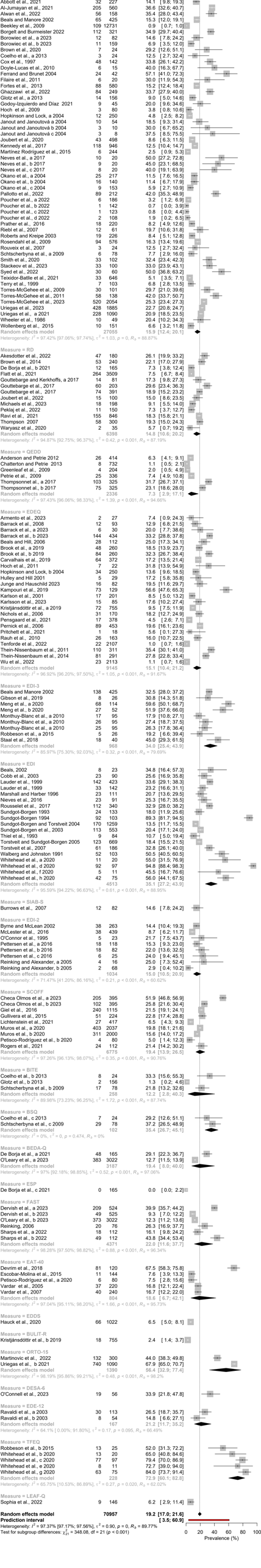

Supplement: Supplementary file 13 — Additional file 13 Subgroup meta-analysis by disordered eating measurement tool. [file 40337_2024_982_MOESM13_ESM.pdf]

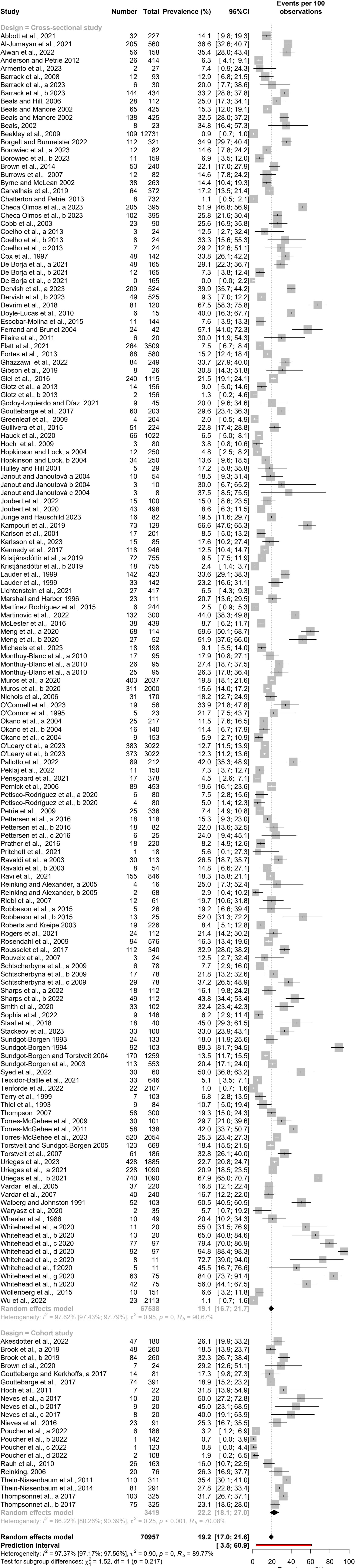

Supplement: Supplementary file 14 — Additional file 14 Subgroup meta-analysis by study design. [file 40337_2024_982_MOESM14_ESM.pdf]
