# Supplementary material for: A systematic review, meta-analysis, and meta-regression of the prevalence of self-reported disordered eating and associated factors among athletes worldwide
Source: J Eat Disord. 2024 Feb 7;12:24. doi: 10.1186/s40337-024-00982-5 (PMC10851573; doi:10.1186/s40337-024-00982-5)

Study	Events	Total	Proportion	95%-CI	Weight (common)	Weight (random)
Abbott et al., 2021	32	227	0.14	[0.10; 0.19]	0.9%	1.6%
Akesdotter et al., 2022	47	180	0.26	[0.20; 0.33]	1.2%	1.8%
Al-Jumayan et al., 2021	205	560	0.37	[0.33; 0.41]	0.0%	0.0%
Alwan et al., 2022	56	158	0.35	[0.28; 0.43]	0.0%	0.0%
Anderson and Petrie 2012	26	414	0.06	[0.04; 0.09]	0.0%	0.0%
Armento et al., 2023	2	27	0.07	[0.01; 0.24]	0.1%	0.2%
Barrack et al., 2008	12	93	0.13	[0.07; 0.21]	0.4%	0.8%
Barrack et al., a 2023	6	30	0.20	[0.08; 0.39]	0.2%	0.4%
Barrack et al., b 2023	144	434	0.33	[0.29; 0.38]	0.0%	0.0%
Beals and Hill, 2006	28	112	0.25	[0.17; 0.34]	0.7%	1.4%
Beals and Manore 2002	65	425	0.15	[0.12; 0.19]	1.8%	2.2%
Beals and Manore 2002	138	425	0.32	[0.28; 0.37]	0.0%	0.0%
Beals, 2002	8	23	0.35	[0.16; 0.57]	0.2%	0.5%
Beekley et al., 2009	109	12731	0.01	[0.01; 0.01]	0.0%	0.0%
Borgelt and Burmeister 2022	112	321	0.35	[0.30; 0.40]	0.0%	0.0%
Borowiec et al., a 2023	12	82	0.15	[0.08; 0.24]	0.3%	0.8%
Borowiec et al., b 2023	11	159	0.07	[0.04; 0.12]	0.0%	0.0%
Brook et al., a 2019	48	260	0.18	[0.14; 0.24]	1.3%	1.9%
Brook et al., b 2019	84	260	0.32	[0.27; 0.38]	0.0%	0.0%
Brown et al., 2014	53	240	0.22	[0.17; 0.28]	1.4%	1.9%
Brown et al., 2020	7	24	0.29	[0.13; 0.51]	0.2%	0.5%
Burrows et al., 2007	12	82	0.15	[0.08; 0.24]	0.3%	0.8%
Byrne and McLean 2002	38	263	0.14	[0.10; 0.19]	1.1%	1.7%
Carvalhais et al., 2019	64	372	0.17	[0.14; 0.21]	1.8%	2.1%
Chatterton and Petrie 2013	8	732	0.01	[0.00; 0.02]	0.0%	0.0%
Checa Olmos et al., a 2023	205	395	0.52	[0.47; 0.57]	0.0%	0.0%
Checa Olmos et al., b 2023	102	395	0.26	[0.22; 0.30]	0.0%	0.0%
Cobb et al., 2003	23	90	0.26	[0.17; 0.36]	0.6%	1.2%
Coelho et al., a 2013	3	24	0.12	[0.03; 0.32]	0.1%	0.3%
Coelho et al., b 2013	8	24	0.33	[0.16; 0.55]	0.2%	0.5%
Coelho et al., c 2013	7	24	0.29	[0.13; 0.51]	0.2%	0.5%
Cox et al., 1997	48	142	0.34	[0.26; 0.42]	0.0%	0.0%
De Borja et al., a 2021	48	165	0.29	[0.22; 0.37]	0.0%	0.0%
De Borja et al., b 2021	12	165	0.07	[0.04; 0.12]	0.0%	0.0%
De Borja et al., c 2021	0	165	0.00	[0.00; 0.02]	0.0%	0.0%
Dervish et al., a 2023	209	524	0.40	[0.36; 0.44]	0.0%	0.0%
Dervish et al., b 2023	49	525	0.09	[0.07; 0.12]	0.0%	0.0%
Devrim et al., 2018	81	120	0.68	[0.58; 0.76]	0.0%	0.0%
Doyle-Lucas et al., 2010	6	15	0.40	[0.16; 0.68]	0.1%	0.3%
Escobar-Molina et al., 2015	11	144	0.08	[0.04; 0.13]	0.0%	0.0%
Ferrand and Brunet 2004	24	42	0.57	[0.41; 0.72]	0.0%	0.0%
Filaire et al., 2011	6	20	0.30	[0.12; 0.54]	0.1%	0.4%
Flatt et al., 2021	264	3509	0.08	[0.07; 0.08]	0.0%	0.0%
Fortes et al., 2013	88	580	0.15	[0.12; 0.18]	2.5%	2.4%
Ghazzawi et al., 2022	84	249	0.34	[0.28; 0.40]	0.0%	0.0%
Gibson et al., 2019	8	26	0.31	[0.14; 0.52]	0.2%	0.5%
Giel et al., 2016	240	1115	0.22	[0.19; 0.24]	6.3%	2.9%
Glotz et al., a 2013	14	156	0.09	[0.05; 0.15]	0.0%	0.0%
Glotz et al., b 2013	2	156	0.01	[0.00; 0.05]	0.0%	0.0%
Godoy-Izquierdo and Díaz 2021	9	45	0.20	[0.10; 0.35]	0.2%	0.6%
Gouttebarge and Kerkhoffs, a 2017	14	81	0.17	[0.10; 0.27]	0.4%	0.9%
Gouttebarge et al., 2017	60	203	0.30	[0.23; 0.36]	0.0%	0.0%
Gouttebarge et al., 2017	74	391	0.19	[0.15; 0.23]	2.0%	2.2%
Greenleaf et al., 2009	4	204	0.02	[0.01; 0.05]	0.0%	0.0%
Gullivera et al., 2015	51	224	0.23	[0.17; 0.29]	1.3%	1.9%
Hauck et al., 2020	66	1022	0.06	[0.05; 0.08]	0.0%	0.0%
Hoch et al., 2011	7	22	0.32	[0.14; 0.55]	0.2%	0.4%
Hoch et al., 2009	3	80	0.04	[0.01; 0.11]	0.0%	0.0%
Hopkinson and Lock, a 2004	12	250	0.05	[0.03; 0.08]	0.0%	0.0%
Hopkinson and Lock, b 2004	34	250	0.14	[0.10; 0.18]	1.0%	1.6%
Hulley and Hill 2001	5	29	0.17	[0.06; 0.36]	0.1%	0.4%
Janout and Janoutová a 2004	10	54	0.19	[0.09; 0.31]	0.3%	0.7%
Janout and Janoutová b 2004	3	10	0.30	[0.07; 0.65]	0.1%	0.2%
Janout and Janoutová c 2004	3	8	0.38	[0.09; 0.76]	0.1%	0.2%
Joubert et al., 2022	15	100	0.15	[0.09; 0.24]	0.4%	1.0%
Joubert et al., 2020	43	498	0.09	[0.06; 0.11]	0.0%	0.0%
Junge and Hauschild 2023	16	82	0.20	[0.12; 0.30]	0.4%	1.0%
Kampouri et al., 2019	73	129	0.57	[0.48; 0.65]	0.0%	0.0%
Karlson et al., 2001	17	201	0.08	[0.05; 0.13]	0.0%	0.0%
Karlsson et al., 2023	15	85	0.18	[0.10; 0.27]	0.4%	1.0%
Kennedy et al., 2017	118	946	0.12	[0.10; 0.15]	0.0%	0.0%
Kristjánsdóttir et al., a 2019	72	755	0.10	[0.08; 0.12]	0.0%	0.0%
Kristjánsdóttir et al., b 2019	18	755	0.02	[0.01; 0.04]	0.0%	0.0%
Lauder et al., 1999	142	423	0.34	[0.29; 0.38]	0.0%	0.0%
Lauder et al., 1999	33	142	0.23	[0.17; 0.31]	0.8%	1.5%
Lichtenstein et al., 2021	27	417	0.06	[0.04; 0.09]	0.0%	0.0%
Marshall and Harber 1996	23	111	0.21	[0.14; 0.29]	0.6%	1.2%
Martínez Rodríguez et al., 2015	6	244	0.02	[0.01; 0.05]	0.0%	0.0%
Martinovic et al., 2022	132	300	0.44	[0.38; 0.50]	0.0%	0.0%
McLester et al., 2016	38	439	0.09	[0.06; 0.12]	0.0%	0.0%
Meng et al., a 2020	68	114	0.60	[0.50; 0.69]	0.0%	0.0%
Meng et al., b 2020	27	52	0.52	[0.38; 0.66]	0.0%	0.0%
Michaels et al., 2023	18	198	0.09	[0.05; 0.14]	0.0%	0.0%
Monthuy-Blanc et al., a 2010	17	95	0.18	[0.11; 0.27]	0.5%	1.0%
Monthuy-Blanc et al., a 2010	26	95	0.27	[0.19; 0.37]	0.6%	1.3%
Monthuy-Blanc et al., a 2010	25	95	0.26	[0.18; 0.36]	0.6%	1.3%
Muros et al., a 2020	403	2037	0.20	[0.18; 0.22]	10.8%	3.1%
Muros et al., b 2020	311	2000	0.16	[0.14; 0.17]	8.8%	3.1%
Neves et al., a 2017	10	20	0.50	[0.27; 0.73]	0.0%	0.0%
Neves et al., b 2017	9	20	0.45	[0.23; 0.68]	0.0%	0.0%
Neves et al., c 2017	8	20	0.40	[0.19; 0.64]	0.2%	0.4%
Nichols et al., 2006	31	170	0.18	[0.13; 0.25]	0.8%	1.5%
Nieves et al., 2016	23	91	0.25	[0.17; 0.35]	0.6%	1.2%
O'Connell et al., 2023	19	56	0.34	[0.22; 0.48]	0.0%	0.0%
O'Connor et al., 1995	5	23	0.22	[0.07; 0.44]	0.1%	0.4%
Okano et al., a 2004	25	217	0.12	[0.08; 0.17]	0.0%	0.0%
Okano et al., b 2004	16	140	0.11	[0.07; 0.18]	0.5%	1.1%
Okano et al., c 2004	9	153	0.06	[0.03; 0.11]	0.0%	0.0%
O'Leary et al., a 2023	383	3022	0.13	[0.12; 0.14]	0.0%	0.0%
O'Leary et al., b 2023	373	3022	0.12	[0.11; 0.14]	0.0%	0.0%
Pallotto et al., 2022	89	212	0.42	[0.35; 0.49]	0.0%	0.0%
Peklaj et al., 2022	11	150	0.07	[0.04; 0.13]	0.0%	0.0%
Pensgaard et al., 2021	17	378	0.04	[0.03; 0.07]	0.0%	0.0%
Pernick et al., 2006	89	453	0.20	[0.16; 0.24]	2.4%	2.4%
Petisco-Rodríguez et al., a 2020	6	80	0.07	[0.03; 0.16]	0.0%	0.0%
Petisco-Rodríguez et al., b 2020	4	80	0.05	[0.01; 0.12]	0.0%	0.0%
Petrie et al., 2009	25	336	0.07	[0.05; 0.11]	0.0%	0.0%
Pettersen et al., a 2016	18	118	0.15	[0.09; 0.23]	0.5%	1.1%
Pettersen et al., b 2016	18	82	0.22	[0.14; 0.32]	0.5%	1.0%
Pettersen et al., c 2016	6	25	0.24	[0.09; 0.45]	0.2%	0.4%
Poucher et al., a 2022	6	186	0.03	[0.01; 0.07]	0.0%	0.0%
Poucher et al., b 2022	1	142	0.01	[0.00; 0.04]	0.0%	0.0%
Poucher et al., c 2022	1	123	0.01	[0.00; 0.04]	0.0%	0.0%
Poucher et al., d 2022	2	108	0.02	[0.00; 0.07]	0.0%	0.0%
Prather et al., 2016	18	220	0.08	[0.05; 0.13]	0.0%	0.0%
Pritchett et al., 2021	1	18	0.06	[0.00; 0.27]	0.0%	0.1%
Rauh et al., 2010	26	163	0.16	[0.11; 0.22]	0.7%	1.4%
Ravaldi et al., a 2003	30	113	0.27	[0.19; 0.36]	0.7%	1.4%
Ravaldi et al., b 2003	8	54	0.15	[0.07; 0.27]	0.2%	0.6%
Ravi et al., 2021	155	846	0.18	[0.16; 0.21]	4.2%	2.7%
Reinking and Alexander, a 2005	4	16	0.25	[0.07; 0.52]	0.1%	0.3%
Reinking and Alexander, b 2005	2	68	0.03	[0.00; 0.10]	0.0%	0.0%
Reinking, 2006	20	76	0.26	[0.17; 0.38]	0.5%	1.1%
Riebl et al., 2007	12	61	0.20	[0.11; 0.32]	0.3%	0.8%
Robbeson et al., a 2015	5	26	0.19	[0.07; 0.39]	0.1%	0.4%
Robbeson et al., b 2015	13	25	0.52	[0.31; 0.72]	0.0%	0.0%
Roberts and Reinking 2003	19	226	0.08	[0.05; 0.13]	0.0%	0.0%
Rogers et al., 2021	24	112	0.21	[0.14; 0.30]	0.6%	1.3%
Rosendahl et al., 2009	94	576	0.16	[0.13; 0.20]	2.6%	2.4%
Rousselet et al., 2017	112	340	0.33	[0.28; 0.38]	0.0%	0.0%
Rouveix et al., 2007	3	24	0.12	[0.03; 0.32]	0.1%	0.3%
Schtscherbyna et al., a 2009	6	78	0.08	[0.03; 0.16]	0.0%	0.0%
Schtscherbyna et al., b 2009	17	78	0.22	[0.13; 0.33]	0.4%	1.0%
Schtscherbyna et al., c 2009	29	78	0.37	[0.26; 0.49]	0.0%	0.0%
Sharps et al., a 2022	18	112	0.16	[0.10; 0.24]	0.5%	1.1%
Sharps et al., b 2022	49	112	0.44	[0.34; 0.53]	0.0%	0.0%
Smith et al., 2020	33	102	0.32	[0.23; 0.42]	0.0%	0.0%
Sophia et al., 2022	9	146	0.06	[0.03; 0.11]	0.0%	0.0%
Staal et al., 2018	18	40	0.45	[0.29; 0.62]	0.0%	0.0%
Stackeov et al., 2023	33	100	0.33	[0.24; 0.43]	0.0%	0.0%
Sundgot-Borgen 1993	24	133	0.18	[0.12; 0.26]	0.7%	1.3%
Sundgot-Borgen 1994	92	103	0.89	[0.82; 0.95]	0.0%	0.0%
Sundgot-Borgen and Torstveit 2004	170	1259	0.14	[0.12; 0.16]	0.0%	0.0%
Sundgot-Borgen et al., 2003	113	553	0.20	[0.17; 0.24]	3.0%	2.5%
Syed et al., 2022	30	60	0.50	[0.37; 0.63]	0.0%	0.0%
Teixidor-Batlle et al., 2021	33	646	0.05	[0.04; 0.07]	0.0%	0.0%
Tenforde et al., 2022	22	2107	0.01	[0.01; 0.02]	0.0%	0.0%
Terry et al., 1999	7	103	0.07	[0.03; 0.14]	0.0%	0.0%
Thein-Nissenbaum et al., 2011	110	311	0.35	[0.30; 0.41]	0.0%	0.0%
Thein-Nissenbaum et al., 2014	81	291	0.28	[0.23; 0.33]	0.0%	0.0%
Thiel et al., 1993	9	84	0.11	[0.05; 0.19]	0.3%	0.7%
Thompson 2007	58	300	0.19	[0.15; 0.24]	1.6%	2.0%
Thompsonnet al., a 2017	103	325	0.32	[0.27; 0.37]	0.0%	0.0%
Thompsonnet al., b 2017	75	325	0.23	[0.19; 0.28]	1.9%	2.2%
Torres-McGehee et al., 2009	30	101	0.30	[0.21; 0.40]	0.7%	1.4%
Torres-McGehee et al., 2011	58	138	0.42	[0.34; 0.51]	0.0%	0.0%
Torres-McGehee et al., 2023	520	2054	0.25	[0.23; 0.27]	0.0%	0.0%
Torstveit and Sundgot-Borgen 2005	123	669	0.18	[0.16; 0.22]	3.4%	2.6%
Torstveit et al., 2007	61	186	0.33	[0.26; 0.40]	0.0%	0.0%
Uriegas et al., 2023	428	1885	0.23	[0.21; 0.25]	11.1%	3.1%
Uriegas et al., a 2021	228	1090	0.21	[0.19; 0.23]	6.0%	2.9%
Uriegas et al., b 2021	740	1090	0.68	[0.65; 0.71]	0.0%	0.0%
Vardar et al., 2005	37	220	0.17	[0.12; 0.22]	1.0%	1.7%
Vardar et al., 2007	40	240	0.17	[0.12; 0.22]	1.1%	1.7%
Walberg and Johnston 1991	52	103	0.50	[0.40; 0.60]	0.0%	0.0%
Waryasz et al., 2020	2	35	0.06	[0.01; 0.19]	0.1%	0.2%
Wheeler et al., 1986	10	49	0.20	[0.10; 0.34]	0.3%	0.7%
Whitehead et al., a 2020	11	20	0.55	[0.32; 0.77]	0.0%	0.0%
Whitehead et al., b 2020	13	20	0.65	[0.41; 0.85]	0.0%	0.0%
Whitehead et al., c 2020	77	97	0.79	[0.70; 0.87]	0.0%	0.0%
Whitehead et al., d 2020	92	97	0.95	[0.88; 0.98]	0.0%	0.0%
Whitehead et al., e 2020	8	11	0.73	[0.39; 0.94]	0.0%	0.0%
Whitehead et al., f 2020	5	11	0.45	[0.17; 0.77]	0.1%	0.3%
Whitehead et al., g 2020	63	75	0.84	[0.74; 0.91]	0.0%	0.0%
Whitehead et al., h 2020	42	75	0.56	[0.44; 0.67]	0.0%	0.0%
Wollenberg et al., 2015	10	151	0.07	[0.03; 0.12]	0.0%	0.0%
Wu et al., 2022	23	2113	0.01	[0.01; 0.02]	0.0%	0.0%
**Common effect model**		**70957**	**0.19**	**[0.19; 0.20]**	**100.0%**	**--**
**Random effects model**			**0.20**	**[0.19; 0.21]**	**--**	**100.0%**
**Prediction interval**				**[0.15; 0.26]**		

Heterogeneity: $I^2 = 53\%$, $\tau^2 = 0.0312$, $p < 0.01$

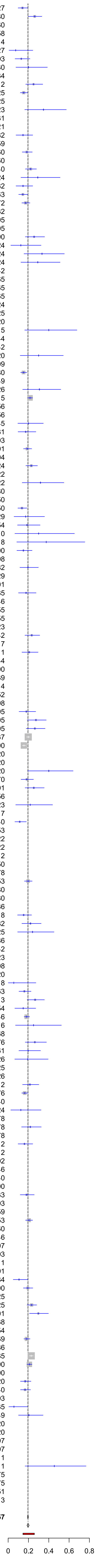

Supplement: Supplementary file 5 — Additional file 5 Outlier analysis. [file 40337_2024_982_MOESM5_ESM.pdf]
